# Prior fear learning enables the rapid assimilation of new fear memories directly into cortical networks

**DOI:** 10.1371/journal.pbio.3001789

**Published:** 2022-09-30

**Authors:** Giulia Concina, Annamaria Renna, Luisella Milano, Benedetto Sacchetti

**Affiliations:** Rita Levi-Montalcini Department of Neuroscience, University of Turin, Turin, Italy; Institute of Science and Technology Austria, AUSTRIA

## Abstract

Long-term memory formation involves the reorganization of brain circuits, termed system consolidation. Whether and how a prior fear experience influences system consolidation of new memories is poorly understood. In rats, we found that prior auditory fear learning allows the secondary auditory cortex to immediately encode new auditory memories, with these new memories purely requiring the activation of cellular mechanisms of synaptic consolidation within secondary auditory cortex. Similar results were obtained in the anterior cingulate cortex for contextual fear memories. Moreover, prior learning enabled connections from these cortices to the basolateral amygdala (BLA) to support recent memory retention. We propose that the reorganization of circuits that characterizes system consolidation occurs only in the first instance that an event is learned, subsequently allowing the immediate assimilation of new analogous events in final storage sites.

## Abstract 2

This study reveals that the occurrence of a prior fear experience allows new memories to be stored in the cortex immediately, without the need of the prolonged reorganization of brain circuits that characterizes the system memory consolidation.

## Introduction

New memories undergo a prolonged process of stabilization or “consolidation” in order to be maintained for a long time. Memory consolidation occurs at both the synaptic and system levels [1–3]. Synaptic consolidation entails changes in synaptic transmission and strength, and it is normally achieved within minutes to hours after learning. System consolidation refers to a time-dependent reorganization of brain circuits that support memory, and it is usually thought to take hours and days in rodents and weeks to months in humans. The standard model of system consolidation was originally proposed for hippocampal-dependent memories and assumes that the formation and retrieval of new memories initially require the hippocampus. Over time and likely through a recurrent interplay between the hippocampus and neocortex, circuits that support memory reorganize so that ultimately a distributed cortical network can store and retrieve remote memories [1–5]. This idea has been demonstrated also in hippocampal-dependent fear memories, namely contextual fear memories, that are initially dependent on the hippocampus and become progressively dependent on a cortical network that encompasses the anterior cingulate cortex (ACC) and the prefrontal cortex [1,2,6–8].

More recently, the time-dependent reorganization of brain circuits has also been described for hippocampal-independent memories [1,9–15], suggesting that it may represent a general mechanism of memory maintenance over time. For example, the higher order auditory (Te2) [10,13,16,17], prefrontal [9,11,12], and retrosplenial [14,15] cortices are critical for remote but not recent auditory fear memories.

Most previous studies were performed on experimentally naïve animals. However, most memories are built on past experiences. Piaget [18] and Bartlett [19] first suggested that new memories that are related to previous analogous experiences can be assimilated more easily. Similarly, Morris and colleagues showed that, in rats, prior experiences accelerated the consolidation of new spatial memories related to flavor-place associations through their incorporation into an associative “schema” [3,20,21]. Subsequently, it was shown that a prior contextual fear learning also changes the molecular mechanisms that underlie the formation of new memories in the hippocampus [22,23]. However, whether and how prior fear experiences influence the development and dynamics of system-level consolidation of new fear memories is unknown.

In this study, we addressed the involvement of the Te2 and ACC in the consolidation of auditory or contextual memories through the blockade of glutamate ionotropic receptors, the inhibition of protein synthesis, and via optogenetic inactivation in naïve rats and in rats that had previously learned another analogous fear event.

## Results

### The system consolidation of a prior auditory fear learning enables the higher order auditory cortex to be necessary for the formation of new memories

**Fig 1:**
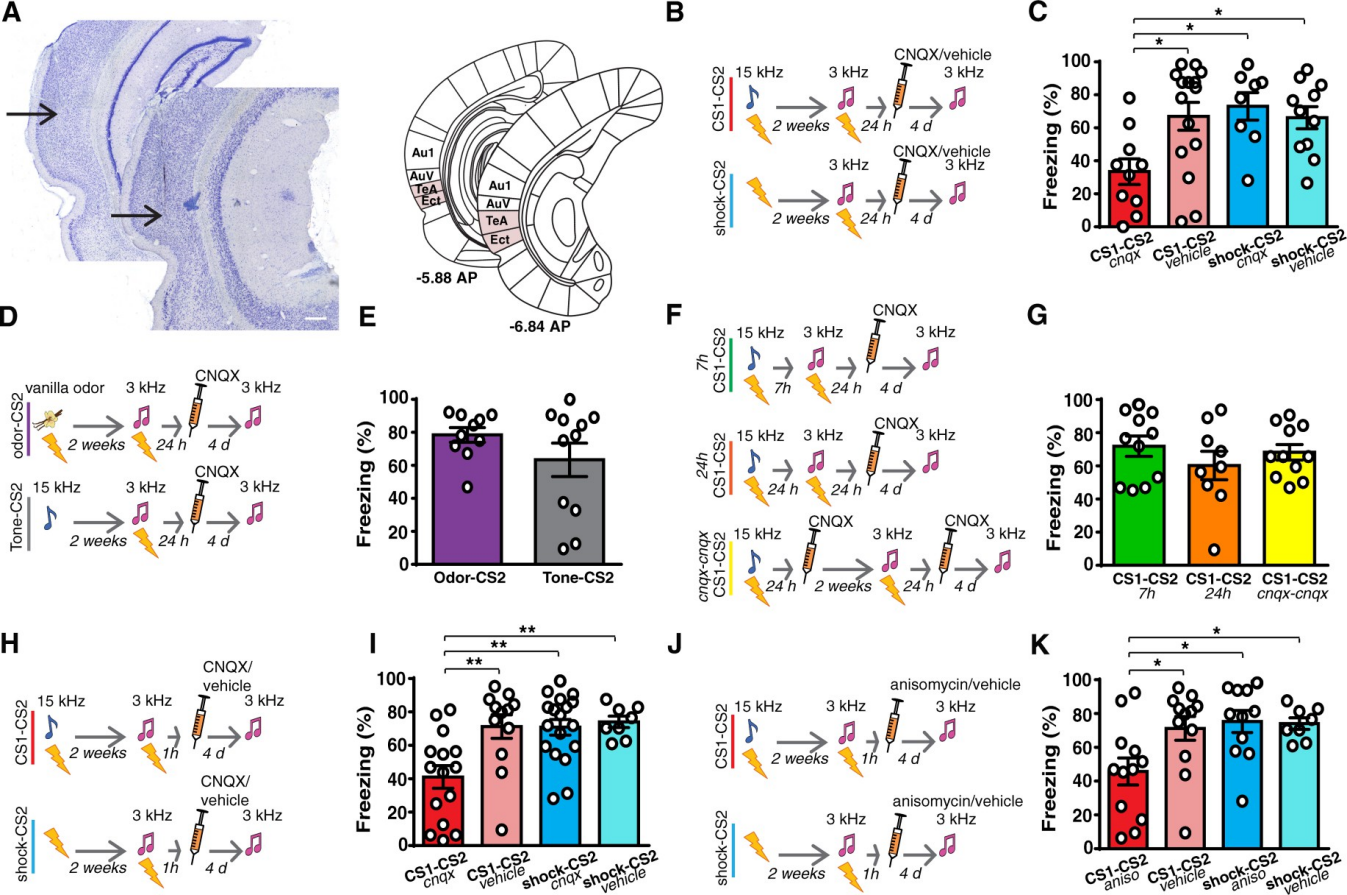
A prior auditory fear learning makes the Te2 necessary for the formation of new, recent memories. ** (A)** Example of micrographs of the needle track in Te2. The arrows indicate the end of the needle track. Based on the Zilles atlas [25], Te2 refers primarily to the temporal association cortex (TeA) described in the Paxinos and Watson atlas [26] and also encompasses the ectorhinal (Ect) cortex. Scale bars, 300 μm. **(B** and **C)** Bilateral CNQX injection into the Te2 24 h after the CS2 (3-kHz)-US pairing impaired the retention of this recent memory in rats that had learned a prior association between a different tone and the US (CS1-CS2, *n* = 10) as compared to animals that had received only painful stimuli (shock-CS2, *n* = 8) and to animals that received saline (CS1-CS2, *n* = 15; shock-CS, *n* = 11) (1-way ANOVA, F_(3,40)_ = 4.367, *p* = 0.0094, η^2^ = 0.246; Tukey: CS1-CS2 _cnqx_ vs. CS1-CS2 _vehicle_, *p* = 0.0208; CS1-CS2 _cnqx_ vs. shock-CS2 _cnqx_, *p* = 0.0183; CS1-CS2 _cnqx_ vs. shock-CS2 _vehicle_, *p* = 0.0401; other comparisons, *p* > 0.05). **(D** and **E)**. CNQX did not affect the retention of recent auditory fear memory in rats that have learned one previous odor-US association (odor-CS2, *n* = 10) or that were presented with the 15-kHz tone alone (Tone-CS2, *n* = 11) (Student *t* test t_(19)_ = 1.319, *p* = 0.2027, Hedges’ g = 2.1213). See also S1 Fig. **(F** and **G)** The injection of CNQX into the Te2 did not affect recent fear memories in rats where the prior auditory fear learning had occurred at 7 h (*n* = 11) or 24 h (*n* = 9) apart from the new CS2-US association and in those where the Te2 was blocked 24 h after both the first and the second CS-US associations (*n* = 11) (1-way ANOVA, F_(2,28)_ = 0.80, *p* = 0.4554, η^2^ = 0.054). **(H** and **I)**. Recent fear memory retention was impaired when CNQX was injected in CS1-CS2 group (*n* = 15) 1 h after learning (shock-CS2 group, *n* = 18) as compared to control groups (CS1-CS2, *n* = 12, shock-CS2, *n* = 8) (1-way ANOVA, F_(3,49)_ = 6.99, *p* = 0.0005, η^2^ = 0.299; Tukey: CS1-CS2 _cnqx_ vs. CS1-CS2 _vehicle_, *p* = 0.0046; CS1-CS2 _cnqx_ vs. shock-CS2 _cnqx_, *p* = 0.0017; CS1-CS2 _cnqx_ vs. shock-CS2 _vehicle_, *p* = 0.0065; other comparisons, *p* > 0.05). (**J** and **K)** A similar result was observed in animals where protein synthesis was inhibited by anisomycin injection (CS1-CS2, *n* = 12; shock-CS2 rats *n* = 11) when compared with the same control groups in Fig 1I (1-way ANOVA, F_(3,39)_ = 4.29, *p* = 0.0104, η^2^ = 0.248; Tukey: CS1-CS2 _aniso_ vs. CS1-CS2 _vehicle_, *p* = 0.0443; CS1-CS2 _aniso_ vs. shock-CS2 _aniso_, *p* = 0.0180; CS1-CS2 _aniso_ vs. shock-CS2 _vehicle_, *p* = 0.0465; other comparisons, *p* > 0.05). *P < 0.05, **P < 0.01, ***P < 0.001. All data are mean and SEM. The summary data for Fig 1 can be found in Supporting information in the file named S1 Data. CS, conditioned stimuli; US, unconditioned stimulus.

We investigated whether and how a prior fear event can influence the consolidation of auditory fear memories at the neocortical network level. We focused on the higher order auditory cortex (Te2) because previous studies demonstrated that in naïve animals this cortex is necessary for remote auditory fear memories but not for recent ones [10,13,16,17]. In mammals, the auditory cortex can be divided into a core region, corresponding to the primary auditory cortex, and a surrounding belt area, whose most posterior part corresponds to the area designated Te2 by Zilles and colleagues [24]. Based on the Zilles atlas [25], Te2 corresponds primarly to the temporal association cortex described in the Paxinos and Watson atlas [26] and also encompasses the ventrally adjacent ectorhinal cortex, while the above ventral temporal area designed by Paxinos and Watson [26] has been included in both primary and secondary auditory fields [10,24–28] (Fig 1A).

Two groups of rats were trained to associate a tone (3-kHz pure-tone conditioned stimulus, CS2) and a mild painful shock (unconditioned stimulus, US). Two weeks before making this association, 1 group of animals learned the association of a different tone (15-kHz pure-tone, CS1) with the US, in a different environment (CS1-CS2 group), while the other group received only immediate painful stimuli in a temporally compressed way (shock-CS2 group), to hamper associative processes and the formation of long-term contextual fear memories (Figs 1A, S1A and S1C and S1 Table) [29,30]. We choose the time interval of 2 weeks based on previous studies that investigated the temporal evolution of engram cells during system memory consolidation at 10 and 14 days after conditioning [7,12,31]. In both groups, Te2 was inactivated 24 h after the CS2 (3-kHz tone)-US association through injection of 6-cyano-7-nitroquinoxaline-2,3-dione (CNQX), which selectively blocks the local α-amino-3-hydroxy-5-methyl-isoxazole-4-propionic acid (AMPA) glutamate receptors. Control groups received saline instead of CNQX. Memory retention was tested 4 days later (Fig 1B). This procedure allows acting specifically on memory consolidation processes without interfering with sensory or motor processes occurring during acquisition or retrieval phases [32–34].

In line with previous findings [10,13,16,17], the recently acquired fear memory was unaffected by cortical inactivation in animals that had not previously learned the other auditory fear association. Surprisingly, rats in the CS1-CS2 group exhibited decreased freezing after Te2 inactivation, suggesting that recent memory retention to the CS2 was significantly impaired in rats that had learned the other, distinct, auditory fear association 2 weeks earlier (Fig 1C). This effect was specifically caused by prior associative learning because it was absent in animals that had experienced only immediate painful stimuli (Fig 1C). These results were obtained also by counterbalancing the 2 tones (S1D Fig).

We then investigated whether this phenomenon may be induced also by an auditory experience not related to associative processes or, alternatively, by associative learning but not related to auditory stimuli. Te2 was inactivated 24 h after the CS2 (3-kHz tone)-US association in rats that 2 weeks before received only auditory stimuli (15-kHz pure-tone) not paired to any USs (Tone-CS2 group) or in rats that have learned to associate an olfactory stimulus with the US (odor-CS2 group). In both groups, no amnesic effects were detected (Figs 1D, 1E and S1E). Thus, learning a prior association between a tone and the US, but not perceiving auditory stimuli or USs alone or learning a fear association of a different sensory modality, makes the Te2 necessary for learning new auditory fear memories.

This raised the question of whether the cortical assimilation of recent memories arises specifically through the gradual process of system consolidation, triggered by prior memory, or whether it is simply due to a previously learned fear experience, regardless of whether system consolidation has occurred. We repeated the previous experiment, but with the first and second fear learnings separated from each other by only 7 h, instead of 2 weeks. Moreover, because system consolidation can occur during sleep [1–3], we performed an additional experiment in which the 2 associations were separated from each other by 24 h, to test whether a day–night cycle might be sufficient for cortical circuit reorganization [35]. However, Te2 inactivation did not affect freezing to the recent CS2 that remained high in all groups, thus suggesting that Te2 inactivation did not affect recent memory retention (Fig 1F and 1G).

To assess the requirement of system consolidation for rapid assimilation of new information in the neocortex further, we investigated whether Te2 blockade during system consolidation of the first memory could prevent the subsequent assimilation of the second memory in this cortex. Rats learned 2 fear events, separated by 2 weeks, and CNQX was administered in Te2 24 h after both the first and second learning trials. No significant effects were detected (Fig 1G), suggesting that Te2 inactivation after the first learning event precluded rearrangement of this cortex, and its subsequent involvement in the consolidation of new memories. These results provided converging evidence that fear learning induces a prolonged process of neocortical circuit reorganization that is causally necessary to enable the neocortex to consolidate new memories subsequently.

It is known that fear learning may be accompanied by a fear generalization process, where fear spreads to novel stimuli [36–39]. For such a reason, we pre-exposed rats to the 15-kHz tone before its pairing with the US, a procedure that significantly decreased fear generalization (S2 Fig). Te2 inactivation after the 3-kHz US association induced a decrement in the freezing to this CS2 (S2 Fig). A similar result was obtained by conditioning rats to a white noise (WN) instead of the 15-kHz tone, i.e., a stimulus markedly different from the 3-kHz tone and that did not elicit significant fear generalization (S2 Fig). Thus, prior fear learning enables the neocortex to rapidly encode new memories even in the absence of fear generalization.

### Prior fear learning enables the Te2 to encode new memories immediately and only through cellular mechanisms of synaptic consolidation

Next, we considered how system consolidation of the first memory boosted cortical consolidation of the second memory. Because the Te2 was inactivated 24 h after the second learning, there may be 2 interpretations. System consolidation of the first memory may accelerate within the 24 h the system consolidation of the second memory, as proposed for spatial memories [3,20,21]. Alternatively, system consolidation of the first memory may reorganize cortical circuits so that they can immediately encode new information, without requiring another round of reorganization at the system level. To address this point, we inactivated Te2 1 h after the second learning, i.e., within a period of time that is too short to complete system memory consolidation, even if accelerated (Fig 1H and 1I). Critically, in animals that had previously learned another association, Te2 inactivation impaired recent memory retention (Fig 1I). This short time interval after learning fits better and is normally associated with the time frame of synaptic consolidation [1,2].

This result raised the intriguing possibility that, after prior fear learning, the cortex might encode new memories simply through cellular mechanisms that underlie local synaptic consolidation [1,2]. To test this possibility, anisomycin was injected into Te2 1 h after fear learning. Anisomycin, a broad-spectrum translation inhibitor, is thought to interfere with memory consolidation via inhibition of protein synthesis [1,2], although a recent study showed that it can reduce neural activity [40]. Indeed, CNQX and anisomycin affected memory processes differently [41,42]. Anisomycin caused a significant decrease in freezing to the recent CS2 only in animals that had learned another remote fear event (Fig 1J and 1K). Overall, these data show that, once a fear event has been learned initially, system consolidation triggered by this event enables the cortex to encode subsequent new memories immediately, through the cellular mechanisms involved in the formation of long-term memories locally and that are characteristic of synaptic consolidation.

### Prior learning enables connections from Te2 to the basolateral amygdala to support recent and remote memory retention

The different timescale (min/h versus days/weeks) is only one of the characteristics that distinguish synaptic consolidation from system consolidation. The key feature of the system consolidation model is that neural circuits that support memory undergo time-dependent reorganization, so that brain regions that contribute to the retention of recent memories may differ from those required for remote memories [1–5,9,35]. By showing that system consolidation of a prior memory enables the cortex to be necessary also for recent memories, our results suggest that system consolidation induced by the first learned event may reorganize brain circuits so that the circuits involved in the storage of remote memories can also be involved in the early consolidation of recent fear memories.

**Fig 2:**
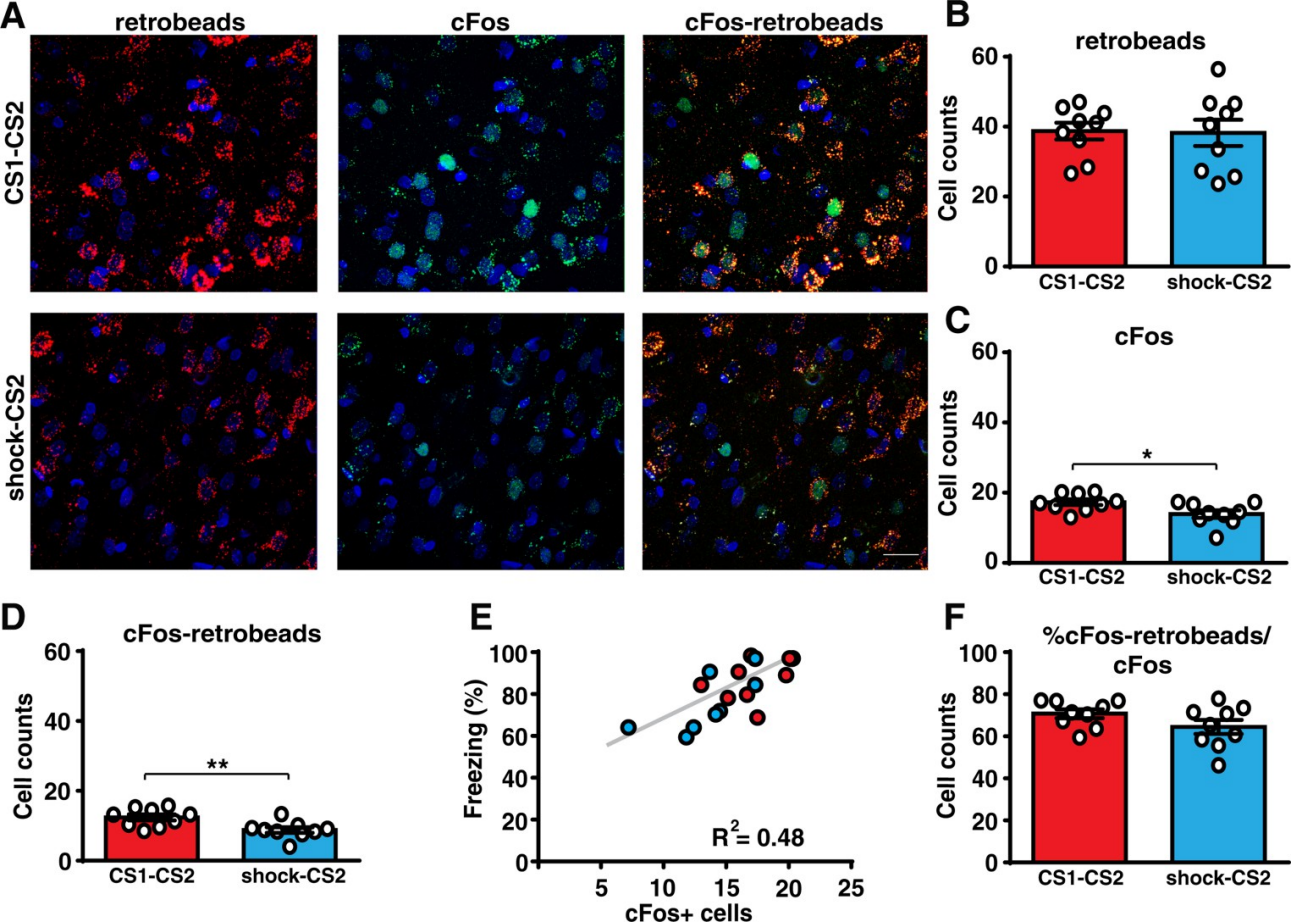
Prior auditory fear learning increases the activity in the Te2-to-BLA pathway. **(A** and **B)** The number of neurons within layer 2/3 of the Te2 projecting to the BLA (retrobeads-labeled) was similar between animals that were trained in two different CS-US associations (CS1-CS2, *n* = 9, upper panel) and in those who received only painful stimuli followed by the CS2-US association 2 weeks later (shock-CS2, *n* = 9, lower panel) (Student *t* test, t_(16)_ = 0.11, *p* = 0.9132, Glass’s delta = 0.006). Scale bars, 20 μm. **(C)** After retention of the recent fear memory, the expression of cFos protein was higher in the CS1-CS2 group (Student *t* test, t_(16)_ = 2.49, *p* = 0.0238, Cohen’s d = 0.117). **(D)** Importantly, the number of cells that project from the Te2 to the BLA and that colocalized with cFos expression was higher in this group (Student *t* test, t_(16)_ = 3.05, *p* = 0.0076, Cohen’s d = 2.99). **(E)** Freezing of CS1-CS2 and shock-CS2 rats to the recent CS correlated with the number of cFos positive cells in Te2 (Pearson’s correlation, r = 0.69, *p* = 0.0013). **(F)** The percentage of double-positive cells on the total cFos positive cells was similar between the 2 groups (Student *t* test, t_(16)_ = 1.59, *p* = 0.1302, Glass’s delta = 0.627). *P < 0.05, **P < 0.01. All data are mean and SEM. The summary data for Fig 2 can be found in Supporting information in the file named S2 Data. BLA, basolateral amygdala; CS, conditioned stimuli; US, unconditioned stimulus.

To test this idea, we analyzed the brain circuit carrying information from Te2 to the basolateral amygdala (BLA). In fact, besides the Te3 and the perirhinal cortex, Te2 represents the major cortical area sending auditory inputs to the BLA. In naïve animals, while the BLA is involved in both recent and remote time intervals, the pathway descending from Te2 to this nucleus is necessary at remote, but not at recent time points [13,17,43]. We traced Te2 projections to the BLA by injecting retrobeads in the BLA of rats trained in the 2 different auditory fear associations, separated by 2 weeks, and in those where the CS2-US association was preceded only by immediate painful stimuli (S3A Fig). Both groups were tested for recent fear memory retention and brains were collected 90 min later. Quantitative blind analysis of Te2-labeled neurons showed a similar number of neurons projecting to the BLA in the 2 groups (Fig 2A and 2B). However, in animals that had learned the 2 different fear associations, there was a higher expression of the activity-dependent protein cFos (Fig 2C), as well as a higher number of double-positive cells that displayed colocalization of cFos expression and the retrograde tracer (Fig 2D). These data showed that the Te2-to-BLA pathway was significantly more activated during the retention of a recently acquired memory in rats that had learned the 2 different associations. Moreover, in both groups the number of cells expressing cFos correlated with the freezing displayed during the recent memory test (Fig 2E). Interestingly, by normalizing the number of cFos and retrolabelled double-positive cells to the total amount of cFos positive neurons, we found no difference between the 2 groups (Fig 2F). This data suggested that, in naïve rats, learning a new association for the first time may activate (or “tag” [44]) the Te2-to-BLA pathway. The subsequent system consolidation process may increase the activity in this pathway so that the subsequent learning of a new association can be supported by a larger number of activated neurons forming this pathway.

**Fig 3:**
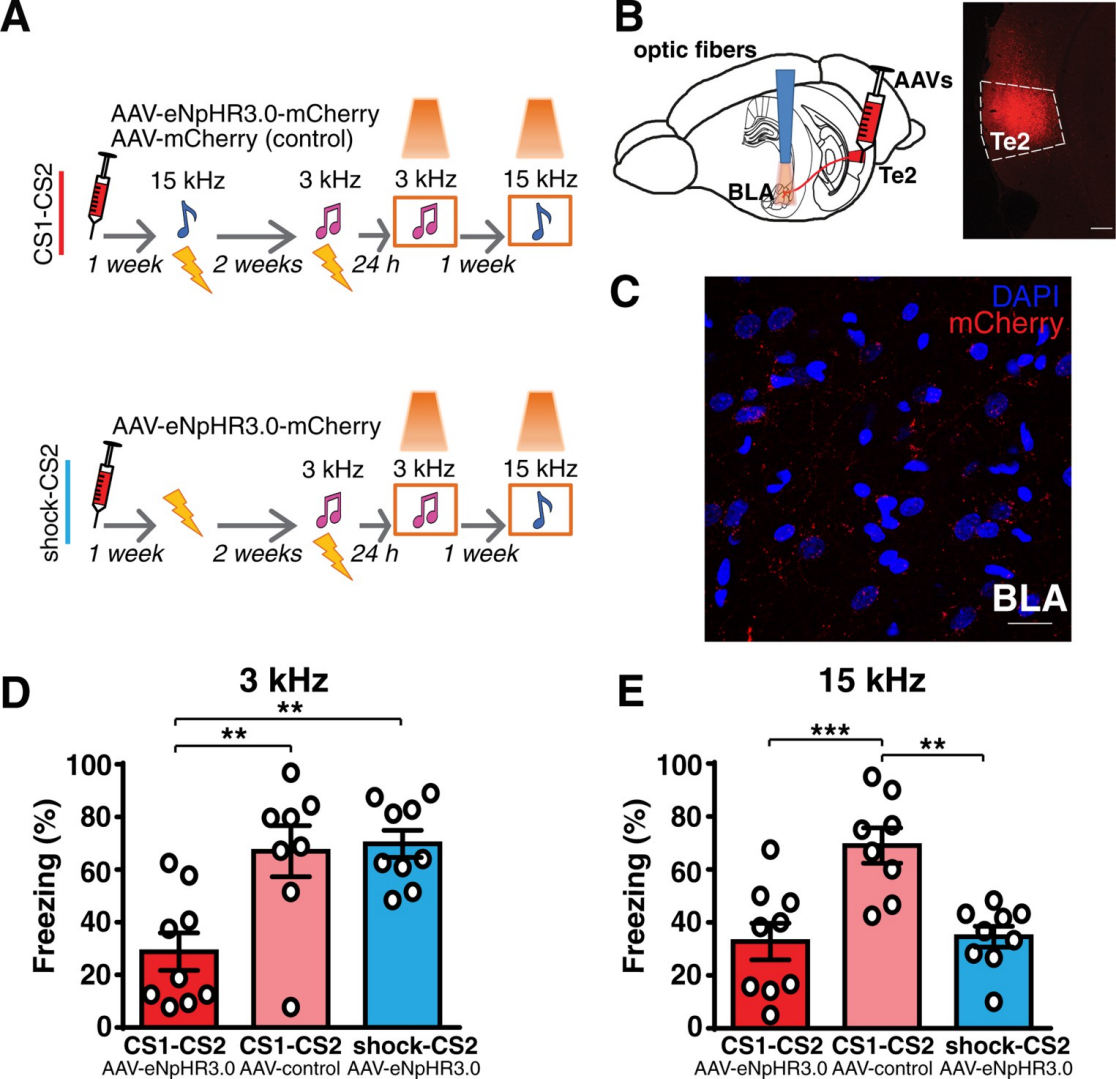
Prior auditory fear learning makes the Te2-to-BLA pathway necessary for the retention of new recent memories. **(A** and **B)** The Te2 was injected with rAAV5/CamKIIa-eNpHR3.0-mCherry-WPRE or rAAV5/CamKIIa-mCherry-WPRE. Optic fibers were implanted above the BLA in order to manipulate Te2 axons terminals within the BLA. **(C)** Representative micrograph showing the expression of AAV-mCherry in Te2 and its terminals in the BLA. Scale bars, 300 and 20 μm, respectively. **(D)** Optogenetic inhibition of Te2 axon terminals in BLA affected the retention of recent fear memory only in animals that had been trained in another CS-US association (1-way ANOVA, F_(2,23)_ = 9.85, *p* = 0.0008, η^2^ = 0.4614; Tukey: CS1-CS2 _eNpHR3.0-mCherry_ *n* = 9 vs. CS1-CS2 _control-mCherry_ *n* = 8, *p* = 0.0040; CS1-CS2 _eNpHR3.0-mCherry_ vs. shock-CS2 _eNpHR3.0-mCherry_ *n* = 9, *p* = 0.0016; CS1-CS2 _control-mCherry_ vs. shock-CS2 _eNpHR3.0-mCherry_, *p* = 0.962). **(E)** Freezing to the tone of 15 kHz was similar between rats in which it had previously been paired to the US (remote fear memory) and in rats that had never been exposed to this tone while it was higher in rats injected with the control vector (1-way ANOVA, F_(2,23)_ = 11.29, *p* = 0.0004, η^2^ = 0.4953; Tukey: CS1-CS2 _eNpHR3.0-mCherry_ vs. CS1-CS2 _control-mCherry_, *p* = 0.0008; CS1-CS2 _eNpHR3.0-mCherry_ vs. shock-CS2 _eNpHR3.0-mCherry_, *p* = 0.972; CS1-CS2 _control-mCherry_ vs. shock-CS2 _eNpHR3.0-mCherry_, *p* = 0.0014), thus suggesting that optogenetic inhibition of the Te2-to-BLA pathway also affected the retention of remote memory. **P < 0.01, ***P < 0.001. All data are mean and SEM. The summary data for Fig 3 can be found in Supporting information in the file named S3 Data. BLA, basolateral amygdala; CS, conditioned stimuli; US, unconditioned stimulus.

To test whether the Te2-to-BLA pathway was causally necessary for recent memory retention, we injected an adeno-associated viral vector (AAV-5) expressing the enhanced light-sensitive chloride pump halorhodopsin eNpHR3.0, combined with red fluorescent protein (mCherry) into the Te2. Expression of the fusion protein was under the control of a CaMKIIα promoter (AAV5-CaMKIIa-eNpHR3.0-mCherry) that drives expression within pyramidal neurons, the neurons that are the major source of efferents to subcortical nuclei [43,45]. Another group of rats received the control vector (AAV5-CaMKIIa-mCherry). Optical fibers were bilaterally implanted above the BLA to inhibit Te2 terminals in the BLA (Figs 3A–3C and S3B) [43]. Rats were trained to learn the CS2 (3-kHz tone)-US association, and 24 h later, we tested recent memory retention. During the retention test, the inhibition of Te2-to-BLA projections caused a significant decrease in the freezing to the CS2 only in rats injected with eNpHR3.0-mCherry vector that had previously learned a distinct tone (15-kHz tone, CS1)-US association (Fig 3A and 3D). Importantly, in these rats, inhibition of this pathway significantly decreased the freezing response during the retention of the remote CS1-US association (Fig 3E). Thus, once animals have formed a new memory for the first time, the neural circuits involved in that memory retention at remote time points are also necessary for the retention of new, subsequently formed memories.

### Prior contextual fear learning enables the anterior cingulate cortex to encode new memories immediately

To date, we have investigated the dynamics of system consolidation in auditory fear memories. However, the model of system consolidation mostly comes from studies on hippocampus-dependent memory. These memories are thought to be formed initially at both the hippocampal and cortical levels. Over time, the coordinated interplay between the hippocampus and cortical networks leads to a gradual remodeling of cortical circuits that eventually store remote memories [1–5]. In line with this, in naïve animals, hippocampal-dependent fear memories, namely contextual fear memories, are initially dependent on the hippocampus and become progressively dependent on a cortical network that encompasses the ACC and the prefrontal cortex [1,2,6–8].

**Fig 4:**
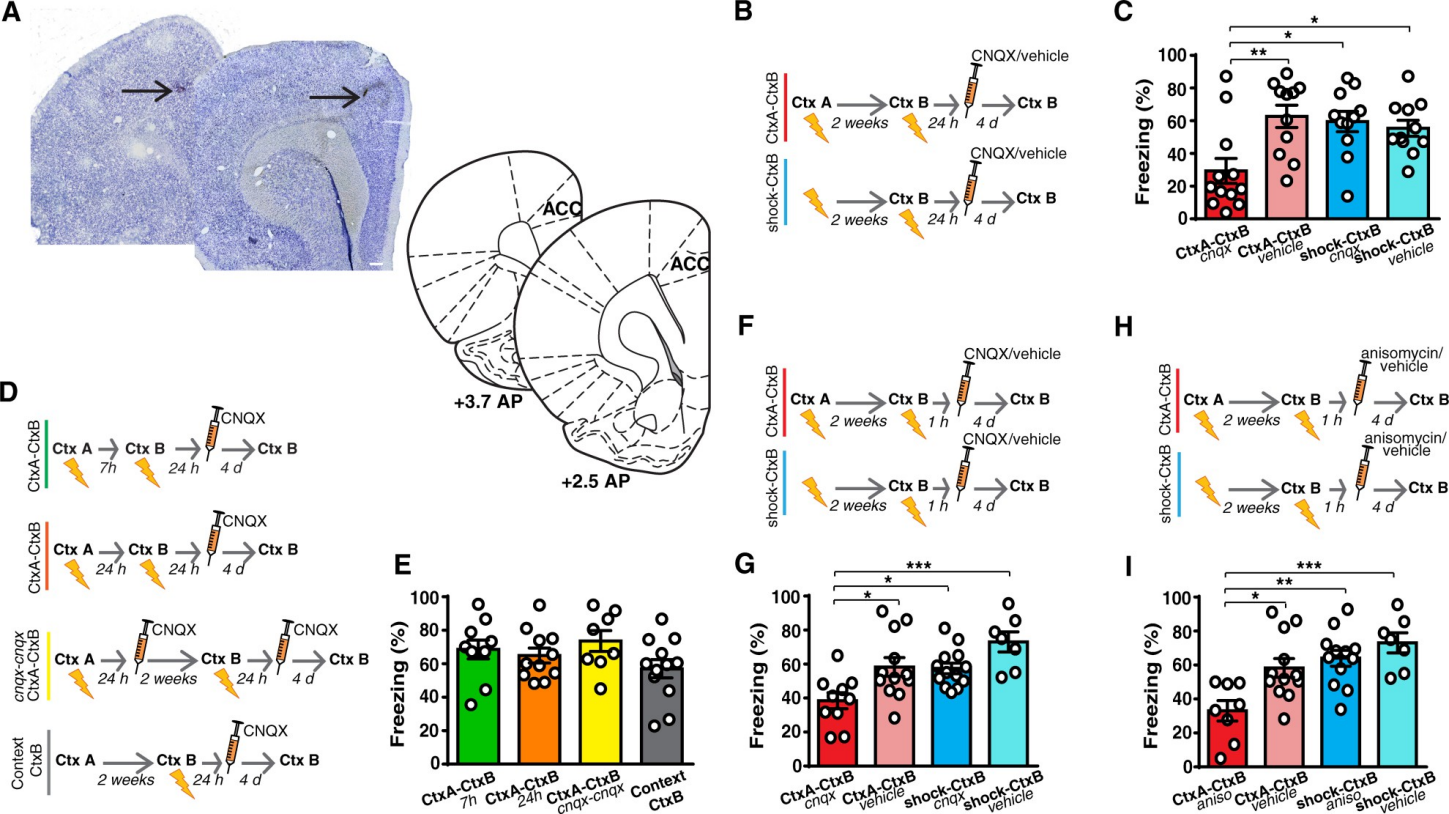
Prior contextual fear learning makes the ACC necessary for the formation of new, recent memories. **(A)** Example of micrograph of needle track in the ACC. Plates are adapted from Paxinos and Watson [26]. Scale bars, 300 μm. **(B** and **C)** Bilateral CNQX injection in the ACC at 24 h after contextual fear learning impaired the retention of recent memory in rats that have learned a prior association between a different context and the US (CtxA-CtxB, *n* = 12) as compared to animals that had received only immediate painful stimuli (shock-CtxB, *n* = 11) or to control rats (CtxA-CtxB, *n* = 11, shock-CtxB, *n* = 11) (1-way ANOVA, F_(3,41)_ = 5.612, *p* = 0.0026, η^2^ = 0.291; Tukey: CtxA-CtxB _cnqx_ vs. CtxA-CtxB _vehicle_, *p* = 0.0041; CtxA-CtxB _cnqx_ vs. shock-CtxB _cnqx_, *p* = 0.0108; CtxA-CtxB _cnqx_ vs. shock-CtxB _vehicle_, *p* = 0.0343; other comparisons, *p* > 0.05). **(D** and **E)** The injection of CNQX in the ACC did not affect recent memories in rats where the prior contextual fear learning occurred at 7 h (*n* = 10) or 24 h (*n* = 11) apart from the new context-US association, in those (*n* = 8) where the ACC was blocked 24 h after both the first and the second contextual fear learning events or in those that explored the remote context in the absence of USs (Context-CtxB, *n* = 12) (1-way ANOVA, F_(3,37)_ = 1.64, *p* = 0.195, η^2^ = 0.117). (**F** and **G**) Recent memories were also impaired when the injection of CNQX was performed 1 h after learning in the CtxA-CtxB group (*n* = 10) as compared to the shock-CtxB group (*n* = 13) and to control rats (CtxA-CtxB group, *n* = 12, shock-CtxB, *n* = 7) (1-way ANOVA, F_(3,38)_ = 7.32, *p* = 0.0005, η^2^ = 0.3663; Tukey: CtxA-CtxB _cnqx_ vs. CtxA-CtxB _vehicle_, *p* = 0.0227; CtxA-CtxB _cnqx_ vs. shock-CtxB _cnqx_, *p* = 0.0276; CtxA-CtxB _cnqx_ vs. shock-CtxB _vehicle_, *p* = 0.0003; other comparisons, *p* > 0.05). (**H** and **I**) Similar results were obtained in rats where protein synthesis was inhibited through anisomycin injection into the ACC (CtxA-CtxB, *n* = 8; shock-CtxB, *n* = 12) when compared with the same control groups in Fig 4G (1-way ANOVA, F_(3,35)_ = 7.820, *p* = 0.0004, η^2^ = 0.4013; Tukey: CtxA-CtxB _aniso_ vs. CtxA-CtxB _vehicle_, *p* = 0.0146; CtxA-CtxB _aniso_ vs. shock-CtxB _aniso_, *p* = 0.0021; CtxA-CtxB _aniso_ vs. shock-CtxB _vehicle_, *p* = 0.0004; other comparisons, *p* > 0.05). *P < 0.05, **P < 0.01, ***P < 0.001. All data are mean and SEM. The summary data for Fig 4 can be found in Supporting information in the file named S4 Data. ACC, anterior cingulate cortex; US, unconditioned stimulus.

We thus considered whether this time-dependent reorganization occurred differently if system consolidation of a prior memory had previously occurred. Rats were trained to associate the context with the US. Two weeks earlier, 1 group of rats had learned another association between a different context and the US (CtxA-CtxB), while another group received only immediate painful stimuli that hampered contextual fear learning (shock-CtxB) (S1A Fig). Animals were injected with CNQX or saline into the ACC at 24 h after contextual learning (Figs 4A and S1B). In line with previous studies [1,6,8], the inactivation of ACC in animals that had not learned another fear association previously left freezing to the CtxB unaffected, thus suggesting that this manipulation did not affect recent memory. However, similar to the data obtained in auditory fear learning, inactivation of the ACC decreased freezing to the recent CtxB in rats that had learned a prior contextual fear association (Fig 4B and 4C). This phenomenon was causally related to the development of the slow system consolidation of the first memory, because it was absent in animals where the 2 fear events were separated by only 7 or 24 h, and in those where the ACC was inactivated 24 h after both the first and the second contextual fear learning. Moreover, it was absent in rats that experienced the exploration of the first context in the absence of any USs (Context-CtxB group) (Fig 4D and 4E).

Critically, inactivation of the ACC performed 1 h after learning also affected recent fear memory (Fig 4F and 4G). At this time point, anisomycin administration decreased freezing to the CtxB in animals that had experienced prior contextual fear learning (Fig 4H and 4I). These data support the idea that, once a first fear event has been memorized, the neocortex becomes essential for new memories immediately, by employing only the synaptic consolidation mechanisms.

### Prior contextual fear learning reorganizes ACC-to-BLA projections so that they support recent memories

**Fig 5:**
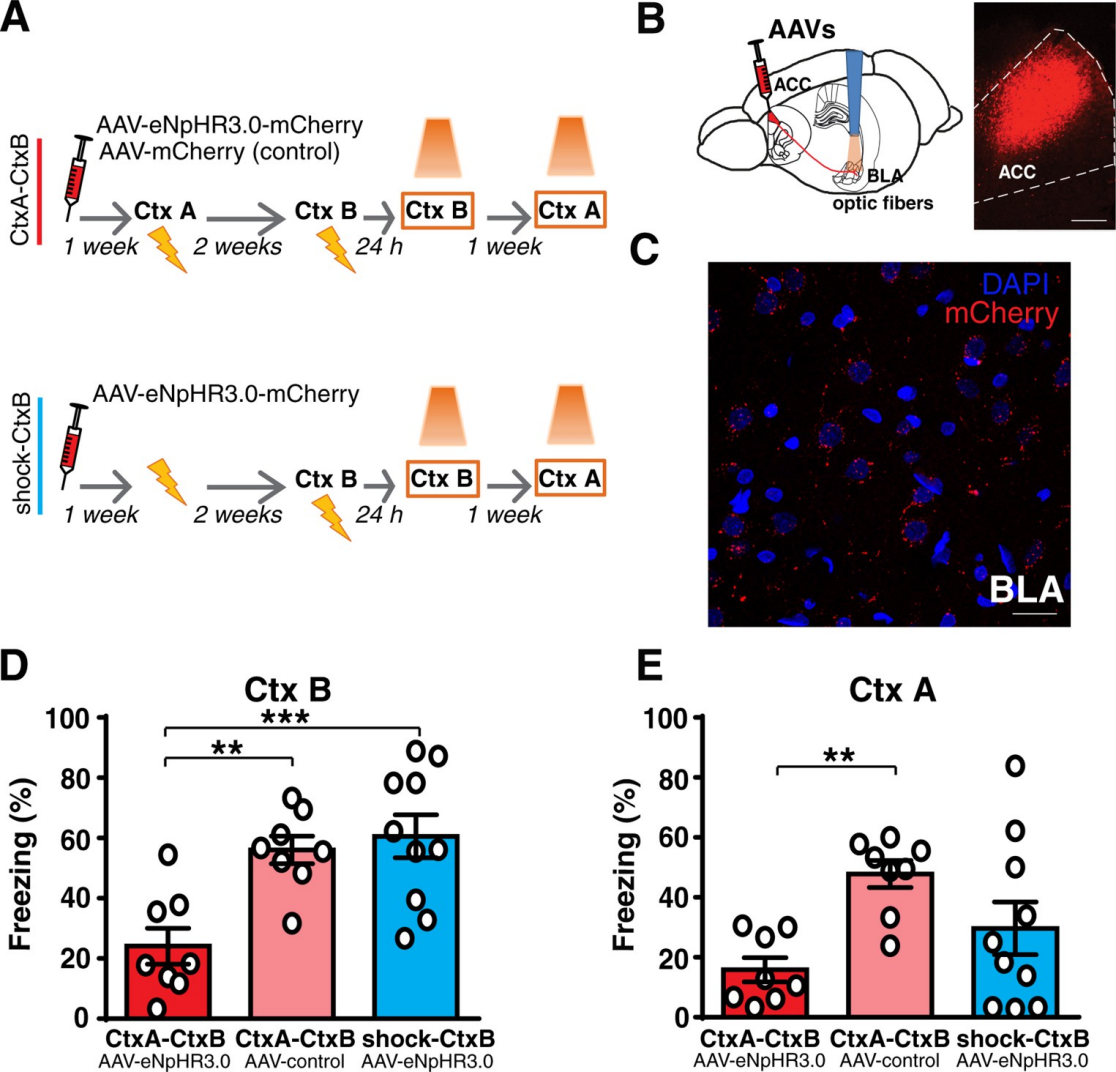
Prior contextual fear learning makes the ACC-to-BLA connectivity necessary for recent memory. **(A** and **B)** To investigate the involvement of the ACC descending pathway to the BLA in the retention of recent and remote contextual fear memories, the ACC was injected with rAAV5/CamKIIa-eNpHR3.0-mCherry-WPRE or rAAV5/CamKIIa-mCherry-WPRE. Optic fibers were implanted above the BLA. **(C)** Representative micrograph showing the expression of AAV-mCherry in the ACC and its terminals in the BLA. Scale bars, 300 and 20 μm, respectively. **(D)** The optogenetic inhibition of ACC axon terminals in the BLA affected the retention of recent fear memory only in animals that had undergone another contextual fear learning previously (1-way ANOVA, F_(2,23)_ = 9.90, *p* = 0.0008, η^2^ = 0.4628; Tukey: CtxA-CtxB _eNpHR3.0-mCherry_ *n* = 8 vs. CtxA-CtxB _control-mCherry_ *n* = 8, *p* = 0.0055; CtxA-CtxB _eNpHR3.0-mCherry_ vs. shock-CtxB _eNpHR3.0-mCherry_ *n* = 10, *p* = 0.0010; CtxA-CtxB _control-mCherry_ vs. shock-CtxB _eNpHR3.0-mCherry_, *p* = 0.8629). **(E)** Freezing to another different context was lower in rats where this context was previously paired to the US (remote fear memory) (1-way ANOVA, F_(2,23)_ = 5.269, *p* = 0.0131, η^2^ = 0.3142; Tukey: CtxA-CtxB _eNpHR3.0-mCherry_ vs. CtxA-CtxB _control-mCherry_ *p* = 0.0098; CtxA-CtxB _eNpHR3.0-mCherry_ vs. shock-CtxB _eNpHR3.0-mCherry_, *p* = 0.3203; CtxA-CtxB _control-mCherry_ vs. shock-CtxB_eNpHR3.0-mCherry_, *p* = 0.1521), thus showing that optogenetic inhibition of the ACC-to-BLA pathway also affected the retention of remote memory. **P < 0.01, ***P < 0.001. All data are mean and SEM. The summary data for Fig 5 can be found in Supporting information in the file named S5 Data. ACC, anterior cingulate cortex; BLA, basolateral amygdala; US, unconditioned stimulus.

We next examined whether the neural connectivity that is recruited during remote memory retention in naïve animals can also participate in the recent memory retention in animals that had previously learned another contextual fear memory. To this end, we investigated the involvement of projections from the ACC to the BLA in the retention of recent and remote contextual memories. A previous study showed that optogenetic inhibition of axon terminals projected from the prefrontal cortex to the BLA impaired contextual fear memory retention at remote but not recent time points in naïve animals [7]. The adeno-associated viral vector (AAV5-CaMKIIa-eNpHR3.0-mCherry) or the control vector (AAV5-CaMKIIa-mCherry) were injected into the ACC in 2 groups, as in the experiments described above (Figs 5A–5C and S3C). Optogenetic inhibition of ACC terminals in the BLA during the recent contextual fear memory retention impaired freezing to the CtxB only in animals that were injected with eNpHR3.0-mCherry vector and that had previously learned a distinct context-US association (Fig 5D). In this group, the subsequent inhibition of ACC terminals in the BLA also caused a significant decrease in freezing to the CtxA, thus suggesting that it also affected the retention of the remote context-US association (Fig 5E). These results support the idea that the same neural pathway is essential for the retention of the remote memory of the first fear event and recent memories of new analogous events. These data also suggest that the rearrangement of cortical pathways descending to the BLA, induced by the first memory, is a common process for both auditory and contextual fear memories.

### After a prior fear learning, the dorsal hippocampus and the ACC are both necessary for the formation of new memories

**Fig 6:**
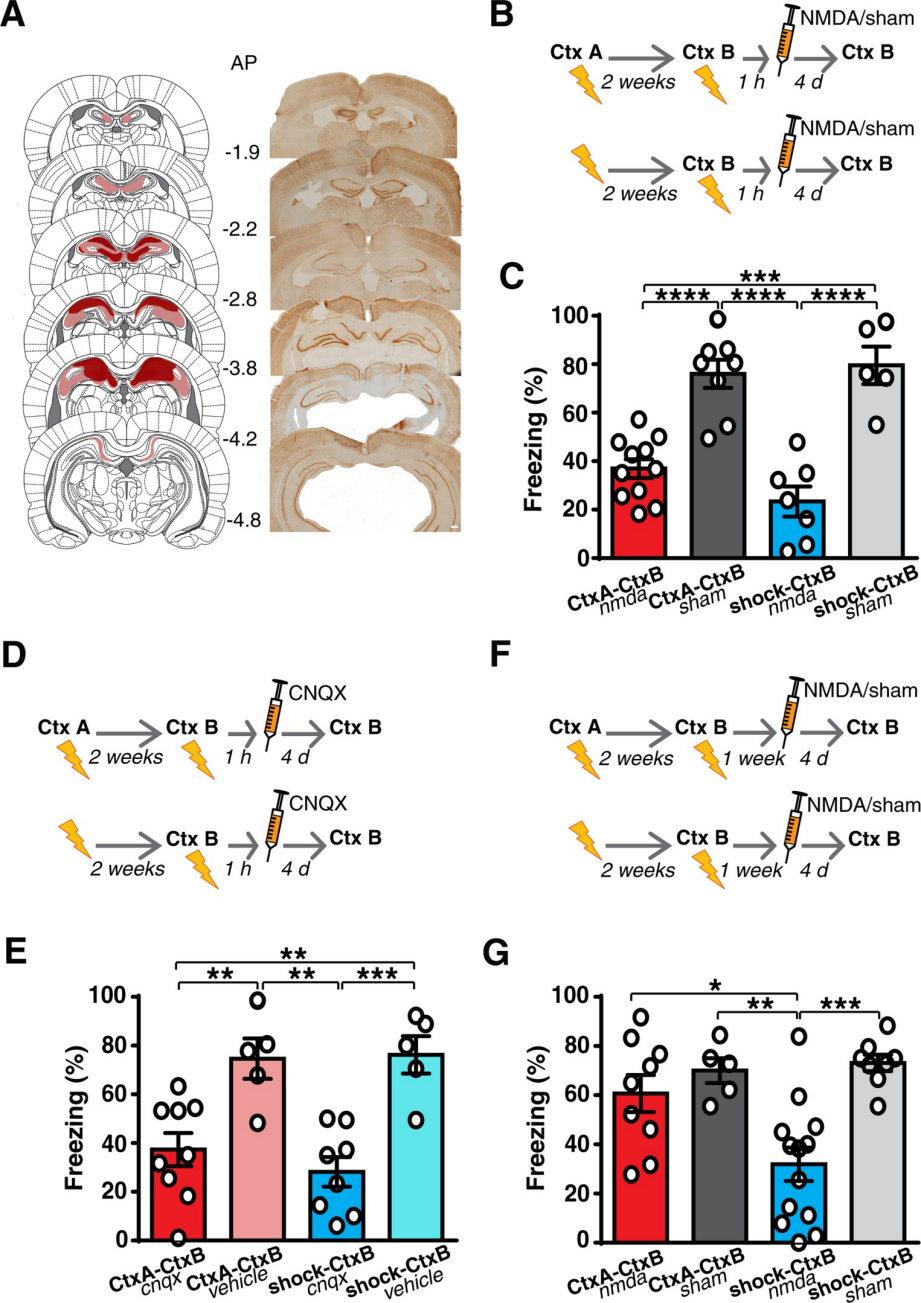
Prior contextual fear learning shortens the time window of hippocampal involvement in learning new associations. **(A–C)** Irreversible lesions of the dorsal hippocampus (CtxA-CtxB, *n* = 11, shock-CtxB, *n* = 7), impaired the formation of new memories in both groups when performed 1 h after learning as compared to sham-lesioned animals (CtxA-CtxB, *n* = 8, shock-CtxB, *n* = 5) (1-way ANOVA, F_(3,27)_ = 23.59, *p* < 0.0001, η^2^ = 0.7238; Tukey: CtxA-CtxB _nmda_ vs. CtxA-CtxB _sham,_ *p* < 0.0001; CtxA-CtxB _nmda_ vs. shock-CtxB _nmda_, *p* = 0.2805; CtxA-CtxB _nmda_ vs. shock-CtxB _sham_, *p* = 0.0001; CtxA-CtxB _sham_ vs. shock-CtxB _nmda_, *p* < 0.0001; CtxA-CtxB _sham_ vs. shock-CtxB _sham_, *p* = 0.9776; shock-CtxB _nmda_ vs. shock-CtxB _sham_, *p* < 0.0001). Histological reconstruction of the excitotoxic lesions aimed at the dorsal hippocampus. Red and pink areas represent the smallest and the largest extension of the lesions. Numbers indicate posterior distance from bregma. Scale bar, 300 μm. (**A**). **(D** and **E)** Similar results were obtained with reversible blockade of the hippocampus (CtxA-CtxB, *n* = 9, shock-CtxB, *n* = 8) with respect saline-injected rats (CtxA-CtxB, *n* = 5, shock-CtxB, *n* = 5) (1-way ANOVA, F_(3,23)_ = 11.24, *p* < 0.0001, η^2^ = 0.5945; Tukey: CtxA-CtxB _cnqx_ vs. CtxA-CtxB _vehicle_, *p* = 0.0078; CtxA-CtxB _cnqx_ vs. shock-CtxB _cnqx_, *p* = 0.7442; CtxA-CtxB_cnqx_ vs. shock-CtxB_vehicle_, *p* = 0.0054; CtxA-CtxB _vehicle_ vs. shock-CtxB _cnqx_, *p* = 0.0012; CtxA-CtxB _vehicle_ vs. shock-CtxB _vehicle_, *p* = 0.9992; shock-CtxB _cnqx_ vs. shock-CtxB _vehicle_, *p* = 0.0008). **(F** and **G)** When performed 1 week after the second learning event, hippocampal lesion did not impair the retention of recent fear memory in animals exposed to both contextual fear conditioning (CtxA-CtxB_nmda_, *n* = 9) (CtxA-CtxB_sham_, *n* = 5), while it impaired recent memory in the shock-CtxB lesioned rats (*n* = 13) (1-way ANOVA, F_(3,31)_ = 9.06, *p* = 0.0002, η^2^ = 0.4673; Tukey: CtxA-CtxB _nmda_ vs. CtxA-CtxB _sham_, *p* = 0.8383; CtxA-CtxB _nmda_ vs. shock-CtxB _nmda_, *p* = 0.012; CtxA-CtxB_nmda_ vs. shock-CtxB _sham_, *p* = 0.5841; CtxA-CtxB _sham_ vs. shock-CtxB _nmda_, *p* = 0.0056; CtxA-CtxB _sham_ vs. shock-CtxB _sham_, *p* = 0.9927; shock-CtxB _nmda_ vs. shock-CtxB _sham_, *p* = 0.0004). *P < 0.05, **P < 0.01, ***P < 0.001, ****P < 0.0001. All data are mean and SEM. The summary data for Fig 6 can be found in Supporting information in the file named S6 Data.

These findings pose the question of whether the hippocampus forms new memories even if prior contextual fear learning has occurred. Alternatively, the cortex might form new memories, even in the absence of the hippocampus. To answer these questions, we inactivated the hippocampus 1 h after contextual fear learning in 2 groups similar to the above experiments. Because most of the literature on system consolidation comes from studies where the hippocampus was destroyed permanently, we began by irreversibly lesioning the dorsal hippocampus 1 h after learning (Fig 6A–6C). Hippocampal lesions caused a significant decrease in the freezing to the CtxB in both groups (Fig 6C). Because excitotoxic lesions might transiently interfere with the activity of brain regions outside the hippocampus, such as the ACC, we repeated this experiment by inactivating the dorsal hippocampus with CNQX to block local AMPA glutamate receptors and found amnesic effects in both groups (Fig 6D and 6E). These results showed that the hippocampus is necessary for the formation of new contextual memories, regardless of whether prior fear learning had occurred. Together with the above results, these data also indicate that, in animals that have learned a prior contextual fear event, both the hippocampus and the ACC are necessary to form recent fear memories, and neither of these sites alone is able to support memory formation in the absence of the other.

We then investigated the time course of hippocampal involvement by delaying hippocampal lesioning to 1 week after learning. In line with previous findings [46], hippocampal lesions still affected memory formation in animals that had never previously learned other fear associations. Conversely, in the group that had learned a prior fear event, hippocampal lesions did not affect memory retention (Fig 6F and 6G), suggesting that a prior fear memory shortens the time window of hippocampal involvement (from 2 weeks to 1 week), as also observed for spatial learnings [3,20,21].

## Discussion

In the present study, we found that prior system consolidation of an auditory fear memory enables the Te2 to immediately encode a new auditory fear memory via synaptic consolidation. Moreover, the descending projections from the Te2 to BLA that were activated during the consolidation of prior learning become necessary in the formation of new auditory fear memories. Similar results were observed in hippocampus-dependent contextual fear learning. In the latter task, we also found that prior learning shortens the duration of hippocampal involvement in recent contextual fear learning. Taken together, our findings suggest that prior learning reorganizes brain circuits so that new analogous information will be learned immediately in cortical structures.

The current concept of system consolidation of hippocampal-dependent memories assumes that the hippocampus is involved in a time-limited manner and that a concomitant gradual process of brain circuit reorganization occurs over time, so that cortical networks become progressively more important [1–5].

Although alternative theories also exist [47], numerous studies support the temporally limited role of the hippocampus in memory processes in both human and animal models [1–3]. On the contrary, the reorganization of brain circuits that support memory over time has been demonstrated mostly in animal models while evidence in humans is more controversial [3]. Some studies showed that hippocampal activity during memory retrieval in humans decreases progressively over weeks and months, whereas activity in the ventral medial prefrontal region [48], in the temporal neocortex [49], or connectivity among cortical areas [48] increased significantly. On the contrary, more recent studies showed that although hippocampal activity declined as time passed, cortical activity remains stable over time [50] or decreases as well [51]. More consistent evidence about the reorganization of brain circuits that support memory over time was obtained in animals [1–3]. These studies were performed on experimentally naïve animals. By investigating this issue in animals that have formed a previous fear memory, here we found that the hippocampus is necessary for the formation of new memories, regardless of whether prior fear learning had occurred. However, the reorganization of brain circuits triggered by the first learning enables the neocortex to encode new memories immediately, simply through cellular mechanisms of synaptic consolidation. This phenomenon applies to both hippocampal-dependent and -independent fear memories and it was specifically induced by the slow system consolidation triggered by the initial learning event. In fact, it was absent in rats where the 2 learning trials were temporally close to each other (a few hours or 1 day) and in those where cortical inactivation after the first learning event precluded rearrangement of the cortex and its subsequent involvement in the rapid assimilation of new memories. Moreover, once the first fear learning has reorganized the brain connectivity, the pathways descending from the cortex to the BLA are not only necessary for the retention of remote memories as in naïve rats but also for recent ones. We conclude that if an analogous memory has been encoded previously, reorganization of brain circuits had already occurred and may no longer be required for the formation of new memories, which conversely engage the cortex and its interplay with subcortical sites immediately.

Despite the large number of studies demonstrating the time-dependent reorganization of brain circuits in naïve animals, the exact purpose of this process is far to be defined. Because the behavioral responses associated with memory may remain similar over time, it is thought that this process serves to improve memory stability over time [1,2,52]. Based on our findings, we propose that, when a fear event occurs for the first time, brain circuits undergo a prolonged process of reorganization, as described in the system consolidation model. This process may serve to rearrange the neural networks that store fear memory permanently so that they can immediately acquire new analogous information through synaptic consolidation mechanisms. Indeed, this operating mechanism may make new learning more “economical,” because it can reduce the expenditure of resources for acquiring new related information, thereby releasing more resources for learning new, unrelated information.

An important question that arises from our findings is whether prior learning permanently changes neural networks so that similar experiences can be stored in the neocortices immediately, even if they occur at very distant time intervals. Alternatively, it could be that the system is reset to the initial level of naïve animals if new similar experiences do not occur in the near future. Experiments assessing the involvement of the neocortex at increasing time intervals between the first and the second learning will allow clarifying this point.

We also found that the blockade of the neocortex during the early consolidation of a first experience prevented the immediate allocation of new similar experiences to the neocortex (see Figs 1F and 4D). These results suggest that cellular processes occurring immediately after the first learning within the neocortex are necessary for its recruitment in the system memory consolidation process. This idea is in line with the findings that some “tagged” cells are activated within the neocortex upon learning in naïve animals and serve for the storage of remote but not recent memories [7,8,12,44]. Future studies are needed to clarify whether these cells play this role also following prior learning or whether they participate also in the formation of recent memories.

Originally, the model of systems consolidation led to the additional idea that the hippocampus learns new information rapidly whereas the neocortex learns it slowly, but subsequent studies demonstrated that the neocortex is capable also of fast learning processes [3,20,21,53]. Our data showed that 2 different cortices (Te2 and ACC) may learn new information immediately if an analogous event has occurred previously. According to our previous studies [10,16,17,34,43,45], we use the term Te2 to refer to the most posterior region of the belt auditory area that, based on Zilles’s studies [24,25], includes mostly the temporal association and ectorhinal areas. Although we cannot exclude that our results may reflect contributions of drug effects also in surrounding regions, namely the adjacent visual cortex, primary auditory cortex, and perirhinal cortex, the data we obtained in naïve animals suggest that they were primarily due to the inactivation of Te2. In fact, we found that in naïve animals, the Te2 was necessary for remote but not recent auditory memories, while inactivation of the adjacent visual cortex left remote auditory memories unaffected [10]. Conversely, a combined blockade of the primary and Te2 cortices impaired also recent memory [34]. Similarly, the inactivation of the anterior perirhinal cortex impaired fear memories [32,54], while the inactivation of the posterior perirhinal cortex did not affect recent [54] and remote [10] fear memories.

Also in the case of ACC and contextual fear memories, the inactivation of the adjacent primary and secondary motor cortex did not affect recent and remote memories [55], while the inactivation of the adjacent prelimbic cortex impaired both recent and remote memories [11].

Concerning the role that these cortices play in memory processes, Te2 participates in the memorization of the emotional value paired to sounds [10,13,16,56]. This idea has been recently demonstrated also in the higher order visual cortex [57,58]. On the other hand, the ACC may form and store memories but also it may modulate the activity of other cortical areas [1–3,6].

Despite the large number of studies showing that Te2 and ACC are necessary for remote but not recent memories, some recent studies reported that these cortices may be necessary also for the retention of recent auditory [34,59] and contextual [55] fear memories. The precise boundary conditions that may determine the necessity of these cortices for recent memories are poorly understood. One of these studies suggested that the intensity of the emotional experience may play an important role [34], but future studies should better address this issue. Future studies will also need to clarify whether the cells that are activated within the neocortex upon learning in naïve animals, and that serve for the storage of remote but not recent memories [7,8,12,44], also play this role following prior learning, or whether they participate also in the formation of recent memories.

Concerning hippocampal participation in memory processes, here we found that the dorsal hippocampus is necessary for learning new contextual memory even if prior contextual learning has occurred. A similar result was observed by performing a within-subjects design on rats that learned both remote and recent contextual memories [60]. On the other hand, it has been also shown that the cellular mechanisms that mediate the subsequent learning in the hippocampus may differ from those involved during the prior learning trial [22,61].

We also found that in animals that experienced contextual fear for the first time, the hippocampus is necessary also 1 week after learning, in line with a previous finding in naïve animals [46]. Conversely, in the other group, hippocampal lesions did not affect memory retention at this time interval, suggesting that a prior fear learning shortens the time window of hippocampal involvement, as also observed for spatial learning [3,20,21]. Those studies and the present one therefore provide converging evidence that the time window of the temporal amnesia induced by hippocampal lesions depends on the prior learned experiences in both spatial and emotional memories.

In those studies, the authors also proposed that training rats for an extended period of time in a spatial task produces a “mental” schema of knowledge, where new related information can be rapidly assimilated [3,20,21,53]. Although our findings may also be consistent with this idea, it is very difficult to say whether one trial of fear learning can form an associative “schema” of knowledge and whether a new and different event occurring in a totally different environment can be integrated within this schema. Further studies are required to clarify this issue. In any case, given that most memories in humans and other animals are built on past experiences, the brain networks that store remote fear memories for the first time may be those that encode both recent and remote fear memories across the lifetime.

## Methods experimental model and subject details

### Animals

Healthy male Wistar rats (age, 65 to 70 days; weight, 250 to 350 g, wild-type), derived from an internal animals facility breading, were housed 3 per cage with food and water available ad libitum, under a 12 h light/dark cycle (lights on at 7:00 AM) at a constant temperature of 22 ± 1°C. All the experiments were approved by the Italian Ministry of Health (authorization no. 408/2020-PR) and by the local bioethical committee of the University of Turin.

### Experimental design

Data reproducibility was assessed with different replicates (S1 Table). The first CNQX inactivation experiments in Te2 (Fig 1A–1C) were performed in a higher number of replicates because they were the first experiments we conducted and served to test the major hypothesis of our study. Animals were a priori assigned to different behavioral groups in a weight-balanced manner. Male animals from the same brood were randomly assigned to each experimental group. We first addressed the main hypothesis of our study, i.e., the cortical inactivation may differently affect the consolidation of a new fear memory in experimentally naïve rats and in animals that had learned a prior fear event. In the case of statistical differences between these groups, we then eventually performed additional control groups through the injection of saline. A similar approach was applied to the optogenetic experiments, where AAV-control group was performed only after statistical differences between naïve and previously trained rats were detected. These experimental schedules allowed to avoid unnecessary control groups and the use of unnecessary animals, a key issue in the European and Italian legislation on animal experimentation (3 Rs principle).

### Behavioral procedures

All the experiments were conducted during the light phase of the day (8 AM to 4 PM). Animals were transported singularly from the facility to the experimental rooms within different small transparent buckets depending on the experimental demands.

#### Auditory training: First behavioral session

*Auditory fear conditioned animals* (CS1-CS2). In this group, rats were gently taken from their home cage and carried from the housing room to the soundproofed room. Once there, animals were placed inside the conditioning apparatus consisting of a rectangular black cage (35 × 40 cm) equipped with a stainless steel rods grid (1 cm in diameter, spaced 1.5 cm apart) connected to a shock delivery setup. Rats were left undisturbed for 1 min. After this time, 7 conditioned stimuli (CSs) consisting of a pure tone of 15 kHz of frequency (15 s of duration each, 80 dB, 36 s inter trials interval) were administered. The last 1 s of each tone was paired with a painful US (0.5 mA, 1 s). At the end of the conditioning session, rats were brought back to their home cage.

*Shock-only animals* (shock-CS2). In this group, rats were similarly placed inside the same conditioning apparatus. Immediately afterward, each rat was subjected to 7 foot shocks (1 s, 0.5 mA) one immediately after the other. At the end of the stimulation, animals were brought back to their home cage. The time permanence in the conditioning cage was less than 9 s. Previous studies showed that this procedure allows avoiding associative processes between painful stimuli and sensory stimuli [29,30].

*Odor fear conditioned animals* (odor-CS2). In this group, rats were placed inside the same rectangular black cage employed in the above experimental groups and connected to a shock delivery setup. Rats were left undisturbed for 2 min. After this time, 7 CSs consisting of vanilla odors were administered (10 s of duration each, 24 s inter trials interval). The last 1 s of each odor was paired with a painful US (0.5 mA, 1 s). The conditioning module was placed nearby a ventilation source in order to avoid the persistence of the delivered stimuli after their offset. The cage was ensured with an upper grid. Odors were presented using a flow-dilution olfactometer. Clean air (1.5 L/min) was directed to a solenoid valve, which when operated, passed the air to a 15 ml bottle containing 10 ml of vanilla odor.

*Tone-only animals* (Tone-CS2). Rats in this group were placed inside the same black cage and were presented with the 15-kHz tone (7 stimuli, 15 s of duration, 36 s ITI) delivered in the absence of the US.

*Pre-exposed animals* (Tone-CS1-CS2). Rats were placed inside the conditioning cage and, 1 min later, they were presented 20 times with the 15-kHz tone alone, and 24 h later the same tone was paired with the US becoming the CS1.

*White noise fear conditioned animals* (WN-CS2). In this group, rats were placed inside the rectangular black cage and left undisturbed for 1 min. After this time, 7 CSs consisting of a WN (15 s of duration each, 75 dB, 36 s inter trials interval) were administered. The last 1 s of each tone was paired with a painful US (0.5 mA, 1 s). At the end of the conditioning session, rats were brought back to their home cage.

#### Auditory fear learning: Second behavioral training

Two weeks after the procedures described in the above paragraph, animals were trained to *another different auditory fear conditioning task*. Rats were put in a standard skinner box, as in our previous work [10], and left undisturbed for 2 min. After this time, 7 CSs consisting of pure tones of 3 kHz of frequency (8 s of duration each, 80 dB, 22 s inter-trial interval) were delivered. The last 1 s of each tone was paired with a painful US (0.5 mA, 1 s). At the end of the conditioning session, rats were brought back to their home cage.

*Fear memory retention*. The retention of auditory fear memory recently acquired to the CS2 (3 kHz) was tested 4 days later. For optogenetics experiments, the test of recent memory was performed with laser delivery 24 h after the CS2-US learning in analogy to the time interval at which we performed cortical inactivation through the administration of the CNQX (i.e., 24 h after training). Rats were habituated to an apparatus different from that used for conditioning and placed in a different room in order to avoid conditioned fear behavior to contextual cues [10,62]. The new apparatus consisted of a transparent plastic cage with a black painted side and enclosed within a sound-attenuating box equipped with an exhaust fan, which eliminated odorized air from the enclosure and provided background noise of 60 dB. Animals were allowed to explore the cage for 5 min a day during the habituation session. On the day of the fear memory retention test, after 2 min of free exploration, we delivered 4 CS2 of 3 kHz (8 s—22 ITI) not followed by any US.

If required by the experimental demand, rats were then tested for the retention of the remote fear memory, acquired 2 weeks before the second auditory fear conditioning trial. To this aim, 7 days after the fear memory retention test to the 3-kHz tone, animals were put in a novel environment (a black and white striped cage) and then presented with the 15-kHz tone. Four tones were presented at 36 s intervals.

#### Contextual training: First behavioral session

*Contextual fear conditioning group* (CtxA-CtxB). In this group, rats were gently taken from their home cage, placed in a bucket, and carried from the housing room to the soundproofed room. Once there, animals were placed inside the conditioning apparatus consisting of the aforementioned rectangular black cage, equipped with the stainless steel rods grid connected to a shock delivery setup. Rats were left undisturbed for 1 min. After this time, 5 US (0.5 mA, 1 s) were administered with 51 s time intervals. At the end of the session, animals were brought back to their home cage.

*Shock-only group* (shock-CtxB). Rats, once placed inside the conditioning apparatus, immediately received 5 foot shocks (1 s, 0.5 mA) one immediately after the other. The time permanence in the conditioning cage was less than 7 s. Previous studies showed that this procedure allows avoiding associative processes between painful stimuli and sensory stimuli [29,30].

*Context-only animals* (context-CtxB*)*. Rats were placed inside the same black cage employed in the above experiments for 5 min without the delivery of any US.

*New contextual fear conditioning and recent fear memory retention*. Two weeks after the procedures described in the above paragraph, all groups were trained to associate a new contextual environment (the skinner box module, placed in a different room) with a painful US (0.5 mA, 1 s). Each animal was placed inside the new chamber and left undisturbed for 2 min. Then, it was exposed to 5 US separated by intervals of 30 s.

The retention of contextual fear memory was tested 4 days after the fear conditioning procedure by putting rats again in the skinner box chamber for 3 min. For optogenetics experiments, the test of recent memory was performed with laser delivery 24 h after the CtxB-US learning in analogy to the time interval at which we performed cortical inactivation through the administration of the CNQX (i.e, 24 h after training). If required by the experimental demand, rats were then tested for the retention of the remote fear memory by putting animals in the context paired to the US 2 weeks before the new association.

Animals were carried in 2 different buckets to the conditioning chambers according to the different contextual procedures.

*Freezing measure*. In all experimental procedures, the assessment of the fear memory retention was determined as a freezing response [10], analyzed as the complete absence of somatic mobility except for respiratory movements. For each animal, the amount of time (in seconds) spent in freezing was measured offline by 2 independent observers who were blinded to the animal groups.

### Surgical procedures

To administer the selected substances in the target sites, rats were anesthetized with isoflurane: The induction was performed at 4% [vol/vol] in 2 L/min medical air and extended to a continuous exposure at 2% [vol/vol]) when rats were mounted in the stereotaxic apparatus.

An incision of the skull was made, and small burr holes were drilled to allow the penetration of a 28-gauge infusion needle. A 10-μl Hamilton syringe mounted on an infusion pump was used to deliver substances. After infusions, the needle was left in place for an additional 3 min. The incision was then closed with stainless steel wound clips, and the animal was given a subcutaneous injection of the analgesic/anti-inflammatory ketoprofen (2 mg/kg body weight), and the animal was kept warm and under observation until recovery from anesthesia.

As in previous studies [10,32–34], the cannulation of animals was unnecessary, with the active compounds being directly administered stereotaxically. This procedure is advantageous because the surgical trauma inherent to the permanent-cannulating procedure is avoided, thus restricting trauma to a single needle penetration [10,32–34]. Indeed, general anesthesia does not significantly affect memory consolidation [10,32–34]. To ensure that the timing of compounds administration related to the learning trial was accurate so that rats received the selected compounds around 1 h and around 1 day after training, isoflurane anesthesia was induced 5 min before the timing of the planned injection, e.g., at 55 min in rats treated 60 min after training and 23 h and 55 min in animals injected at 24 h after training. Each rat was conditioned and then operated separately. Animals belonging to the different behavioral groups were manipulated in an interleaved way.

The selective inhibitor of AMPA/kainate glutamate receptors CNQX (*6-Cyano-7-nitroquinoxaline-2*,*3-dione*) (Tocris, 10 ng/μl) [20,21] was dissolved in sterile saline solution (NaCl, 0.9%) and adjusted to pH 7.4 with HCl. The protein synthesis inhibitor anisomycin (Merck, 125 μg/μl) was dissolved in equimolar HCl, diluted with sterile saline, and adjusted to pH 7.4 with NaOH. Sterile saline (0.9% NaCl) was used as vehicle control. These substances were bilaterally injected at a rate of 0.1 μl/min and at the volume of 0.5 μl per site at the following stereotaxic coordinates taken from Paxinos and Watson atlas [26], with Te2 cortical field refereed to the Zilles atlas [25]:

Secondary auditory cortex (Te2): 1) AP: −5,8 L: ±7,2 DV: −6. 2) AP: -6,8 L: ±7,2 DV: −6.

Anterior cingulate cortex (ACC): 1) AP: +2,5 L: ±0,6 DV: −2,2. 2) AP: +3,7 L: ±0,6 DV: −2,2.

The volume of injection (0.5 μl per site) was selected based on previous studies where active compounds were injected in Te2 [10,34] and ACC [55,63] in adult rats.

To inactivate the dorsal hippocampus, active compounds were injected at a volume of 0.6 μl per site at the following stereotaxic coordinates:

Dorsal Hippocampus: 1) AP: −2,8 L: ±1,6 DV: −3,3. 2) AP: −4,2 L: ±2,6 DV: −3.

The irreversible lesions of the dorsal hippocampal were induced by administering NMDA (Tocris, 18 μg/μl, dissolved in sterile saline solution, 0,4 μl per site) at a rate of 0.1 μl/min at points at the aforementioned coordinates. The same coordinates were used for saline-injected controls and sham-operated animals.

Red Retrobeads (Lumafluor, 1:2 dilution in saline, 0.6 μl) were injected in BLA according to the following coordinates: AP: −2.8; L: ±5.4; DV: −8.3.

At the end of the experiments, the needle tracks in the case of CNQX, anisomycin, or saline injections or the extension of the lesions in the case of NMDA injections were histologically verified. Rats were deeply anesthetized and intracardially perfused with 4% formaldehyde. Their brains were sectioned at 30 μm on a cryostat. Nissl-stained serial sections were prepared using the conventional procedure and the sections were histologically verified under a microscope magnified at 2.5×.

### Optogenetic experiments

#### Virus injection

The adeno-associated virus AAV5:CaMKIIα::eNpHR3.0-mcherry and the control vector AAV5:CaMKIIα-mcherry were obtained from the University of North Carolina Vector Core (Chapel Hill, North Carolina, United States of America). Viral titer was 5.8 × 10^12 vg/ml for both viruses. The use of CaMKII promoter enables transgene expression favoring pyramidal neurons. Viruses were housed in an −80°C freezer. Viral infusions targeting the Te2 or the ACC were performed at the above stereotaxic coordinates at the volumes of 0.5 μl for each hole. The virus was injected at a rate of 0.1 μl/min, and the needle was left in place for an additional 5 min. Viral injections were performed 4 weeks before optogenetics experiments.

#### Illumination

The optic fibers (Plexon, 200/230 μm core; 10 mm length) were implanted bilaterally in the BLA (AP = −2.8, L = ± 5.4, V = −8.2 mm from bregma). Optogenetic inhibition of Te2 projections to BLA or of ACC projections to BLA was obtained by using the PlexBright Optogenetic Stimulation System coupled to a laser diode (Laserglow Technologies). Yellow light (589 nm) generated passed through an optical fiber. The power density estimated at the tip of the optic fiber was 10 to 15 mW for the illumination of projection sites. During fear memory retention, light emission was initiated 4 s prior to tone onset, persisted throughout the duration of the tone, and was stopped 4 s after the tone offset. Animals belonging to contextual fear conditioning groups received the light stimulation during the entire context exploration (3 min). Rats were familiarized with the patch cord for 2 days before the memory retention trial.

#### Immunohistochemistry

Upon completion of optogenetics experiments, rats were deeply anesthetized and perfused intracardially with 4% PAF in order to examine the diffusion of the virus. The brains were dissected, stored overnight at 4°C, and finally transferred to 30% sucrose. Coronal sections (30 μm) were cut on a cryostat and collected in PBS. Free-floating sections were incubated in a blocking solution for 1 h at RT. Then, they were incubated in primary monoclonal mouse antibody anti mCherry (1:500 dilution, Abcam) in the blocking solution overnight at RT. Subsequently, sections were washed with PBS and incubated for 1 h at RT with secondary fluorescent AlexaFluor-568 anti-mouse antibody (1:600, Invitrogen) diluted in PBS. Sections were washed in PBS, mounted with mounting media containing DAPI (Vector), and cover slipped.

Brains of rats that underwent NMDA injections were similarly collected. Free-floating sections, after several rinses, were incubated with primary monoclonal mouse anti-Neun (1:1,000 dilution, Merck) antibody in the blocking solution overnight at room temperature. Subsequently, sections were washed with PBS and incubated for 1 h at room temperature with biotinylated horse anti-mouse antibody (1:200 dilution, Vector). The avidin-biotin complex (ABC complex 1:100, Vector, 2 h and half of incubation) was coupled to diaminobenzidine (0.03%, Merck) to stain Neun. Sections were then rinsed in PBS and transferred to gelatin-coated slides, dehydrated, and covered with a coverslip.

Te2 sections of retrobeads-injected rats underwent the incubation with primary polyclonal rabbit anti-Fos antibody (1:2,000, Cell Signaling) in the blocking solution overnight at RT. Subsequently, slices were washed with PBS and incubated for 1 h at RT with anti-rabbit Alexa 488 (1:1,000, Invitrogen, in PBS). Finally, immunolabeled sections were washed in PBS, mounted on gelatin-coated slides, and cover slipped with a DAPI-supplemented mounting medium.

### Microscopy

mCherry labeling was examined by using a Zeiss Airyscan confocal microscope: Two lasers were used (405 and 561 nm), each corresponding to the peak emission spectrum for DAPI (Nissl stain for cell nuclei) and Alexa 568 (mCherry), respectively. To analyze the diffusion of the virus at the injection sites, micrographs of Te2 and ACC were acquired as mosaic images (each single was acquired by using a 10× objective). Axon terminals into BLA were analyzed by using a 40× objective as a z-stack of 10 sections, spaced 1 μm apart (159 μm square; zoom fraction, 1.0).

For retrobeads-injected animals that underwent cFos immunolabeling, images were acquired at a 40× magnification (159 μm square; zoom fraction, 1.0) with 3 different lasers, corresponding to the peak emission spectrum for DAPI (Nissl stain for cell nuclei), Alexa-Fluor 488 (cFos), and Texas Red (Retrobeads), respectively. Sections of 0.7 μm were acquired along a 10-μm z-stack. The number of nuclei expressing cFos, beads-labeled, and double-positive (cFos+ beads labeled) was quantified for each animal in the Te2 region at the anteroposterior coordinates from 6.7 to 7.3 mm from the bregma [10]. Data were then averaged in order to produce the mean of each animal and the results were statistically compared. Images of sections with DAB staining of Neun were analyzed using Neurolucida software connected to a microscope via a color CCD camera [10].

### Data analysis and exclusion criteria

The n for each group was established a priori according to our previous studies [10,16,17], previously published works in the field (see as references for ACC [6,22,55] and Te2 [10,59]), and through G-power estimations, according to the following table (Table 1):

**Table 1:** Statistical tests and selected parameters.

Statistical test	Power	α Error probability	Tail	Effect size	Allocation ratio	Non sphericity correction	Total sample size
One-way ANOVA (4 groups)	(1-β) = 0.80	0.05		0.6			36
One-way ANOVA (3 groups)	(1-β) = 0.80	0.05		0.6			30
Student *t* test	(1-β) = 0.80	0.05	2	2	N2/N1 = 1		12
Mixed ANOVA (3 groups)	(1-β) = 0.80	0.05		0.3		1	30

Effect sizes refer to eta squared for ANOVAs while to Cohen’s d for Student *t* test. Eta squared for mixed ANOVA were based on the estimation of the interaction effect.

For each analysis, we estimated a final mean number of 8 to 10 animals for each group. Groups were run with internal controls in the same experimental session (S1 Table). Given the variability of surgical and behavioral procedures, we included a priori a greater number of experimental subjects (up to 15% more) that in some cases met the experimental criteria and were included, thus avoiding an arbitrary exclusion. In the case of hippocampal studies, to assess the effects of NMDA and CNQX injections at distinct time intervals, we balanced the total number of control animals between vehicle and sham-operated rats among the different groups.

Behavioral analysis, histological inspections, and cell counts were performed blindly.

Animals with inadequate localization of the needle track or the lesion in the case of NMDA-irreversible lesions were excluded from data analysis (S1 Table). In pharmacological studies, we excluded 57 animals over a total of 465 animals because of an incorrect needle placement (22/255 for Te2, 31/179 for ACC, and 4/31 for hippocampus). In tracing studies, on 21 animals, we eliminated 3 subjects in which the injection of retrobeads missed the BLA. In Te2 optogenetics studies, over a total of 30 animals, we excluded 1 rat because the placement of the optic fibers was not above the target region, 1 rat because the virus was absent in Te2, and 2 rats because the virus diffusion was respectively less than 4.7% and 5.3% of the Te2 area at the injection sites. In rats included in the statistical analysis, the minimal virus diffusion was 39.6% at the more anterior stereotaxic coordinate and 50.2% at the more posterior stereotaxic coordinate.

Similarly, in the ACC optogenetics experiments, over a total of 32 animals, 2 rats were eliminated because the placement of optic fibers was not above the target region. Two rats were excluded because the virus in ACC was absent, 1 animal because the virus was into the adjacent secondary motor cortex, and 1 rat because the virus diffusion was less than 2.3% of the ACC area at the injection sites. In the remaining rats, the minimal virus diffusion was 33.3% at the more anterior stereotaxic coordinate and 42.2% at the more posterior coordinate.

In the NMDA lesions studies, lesions mostly targeted the CA1 region of the dorsal hippocampus. The minimal (red) and maximal extension (pink) of hippocampal lesions over the entire area of the dorsal hippocampus were respectively 23.2% and 53,5% at the more anterior stereotaxic coordinate and 28.3% and 62.3% at the more posterior coordinate. Over a total of 73 animals, 4 rats were eliminated because the lesion was absent, while additional 3 animals were discarded because the extension of the lesions was less than 5.0% (namely 4.8%, 4.3%, 3.1%) with respect to the hippocampal area at the 2 coordinates.

Area contour analysis was performed through visual inspection and manual quantification using the *region-contour* tool of ZEN 3.0 software. Area values were then normalized on the total area of the region. Stereotaxic coordinates of Te2, ACC, and hippocampus were based on the Paxinos atlas [26] and, in the case of Te2, with cortical field refereed to Zilles atlas [25].

### Statistical analysis

All data are presented as mean ± SEM. All data passed the Levene’s test for equality of variances. Thus, parametric statistics were employed throughout all the experiments. Data from 2 groups were compared using 2-tailed unpaired Student *t* tests. Multiple-group comparisons were assessed using 1-way ANOVA test with Tukey’s post hoc test. To address the between and within groups differences, we computed a 3 × 2 mixed-design ANOVA model with group (CS1-CS2, Tone-CS1-CS2, WN-CS2) as between-subjects variable and condition (before and after recent conditioning +injection) as within-subjects variable. Where the group × condition interaction was significant, we performed a simple main effects analysis and we adjusted each *p* value with the Bonferroni correction. For each mixed ANOVA model, we assessed the Sphericity assumption through Mauchly’s Test of Sphericity. The statistical parameters (i.e., exact value of n for each group, SEM, the statistical test, the effect size, and the exact *p* value) are reported in the legends. For equal samples size, effect sizes for unpaired *t* test were determined by calculating Cohen’s d or Glass’s d according to the similarity of SDs while, for different samples size by using Hedges’g. Correlations between cell counts and freezing were calculated using Pearson’s coefficient. To determine whether the data met the assumptions of the statistical approach, we rejected the null hypothesis at the *P* < 0.05 level. All statistical analyses were performed using SPSS Statistics 22 (IBM).

## Supporting information

S1 Fig Magnification of the needle tracks of Te2 **(A)** and ACC cortex **(B)** injected with CNQX, selected as examples. **(C)** When represented with the same context 2 weeks after the procedure, shock-only animals showed a low fear response, demonstrating that the procedure did not elicit a conditioned freezing to the context where the shock was delivered. **(D)** Similar results as in Fig 1 were obtained by counterbalancing the 2 tones employed as CSs (CS1, 3 kHz and CS2, 15 kHz) (Student *t* test, t_(14)_ = 3.44, *p* = 0.0040, Glass’s d = 4.14). **(E)** In the odor-CS conditioned rats freezing to the odor, 2 weeks after conditioning, was high even if tested in a different environment with respect to the conditioning context, thereby showing that fear was specifically associated with this cue delivery. Scale bars, 300 μm. **P < 0.01. All data are mean and SEM. The summary data for S1 Fig can be found in supporting information in the file named S1 Supporting Figure Data.
(TIF)

S2 Fig CNQX injection impaired the retention of recent auditory fear memories in rats where fear generalization was lowered by a tone alone pre-exposure or by employing a white noise tone as CS1. A 3 × 2 mixed-design ANOVA (main effect of group: *F*_(2,42)_ = 6.478, *p* = 0.004, η^2^ = 0. 236, main effect of condition: *F*_(1,42)_ = 0.161, *p* = 0.691, η^2^ = 0.004, group × condition interaction *F*_(2,42)_ = 3.942, *p* = 0.027, η^2^ = 0.158) showed that freezing to the 3 kHz tone before its association to the US was lower in animals that received the 15 kHz tone pre-exposure before the 15 kHz-US pairing (*n* = 10, *p* = 0.001) or in rats conditioned to a white noise (*n* = 13, *p* = 0.008) as compared to CS1-CS2 animals (*n* = 22) that showed a variable fear generalization response. However, after CS-US learning and cnqx injections in Te2 cortex, recent fear memory was impaired in all groups (*p* > 0.05). Simple main effect within groups (before and after CS-US learning followed by cnqx injection): CS1-CS2, *p =* 0.025; Tone-CS1-CS2, *p =* 0.189; WN-CS2, *p* = 0.347) *P < 0.05, **P < 0.01. All data are mean and SEM. The summary data for S2 Fig can be found in Supporting information in the file named S1 Supporting Figure Data.
(TIF)

S3 Fig **(A)** Random example of retrobeads injection targeting BLA. **(B** and **C)** Examples of optical fiber placements above the BLA of animals injected with AAV vectors in Te2 **(B)** and ACC cortex (**C)**. Scale bars, 500 μm.
(TIF)

S1 Data Raw data of Fig 1. (XLSX)

S2 Data Raw data of Fig 2. (XLSX)

S3 Data Raw data of Fig 3. (XLSX)

S4 Data Raw data of Fig 4. (XLSX)

S5 Data Raw data of Fig 5. (XLSX)

S6 Data Raw data of Fig 6. (XLSX)

S1 Statistical Test Data Statistical tests employed in the experiments. (DOCX)

S1 Supporting Figure Data Raw data of S1–S3 Figs. (XLSX)

S1 Table Number of rats employed in each group during each experimental replication. (DOCX)

## Abbreviations

ACC: anterior cingulate cortex
BLA: basolateral amygdala
CS: conditioned stimuli
US: unconditioned stimulus
WN: white noise
