# Supplementary figures and images for: Prior fear learning enables the rapid assimilation of new fear memories directly into cortical networks
Source: PLoS Biol. 2022 Sep 30;20(9):e3001789. doi: 10.1371/journal.pbio.3001789 (PMC9555644; doi:10.1371/journal.pbio.3001789)

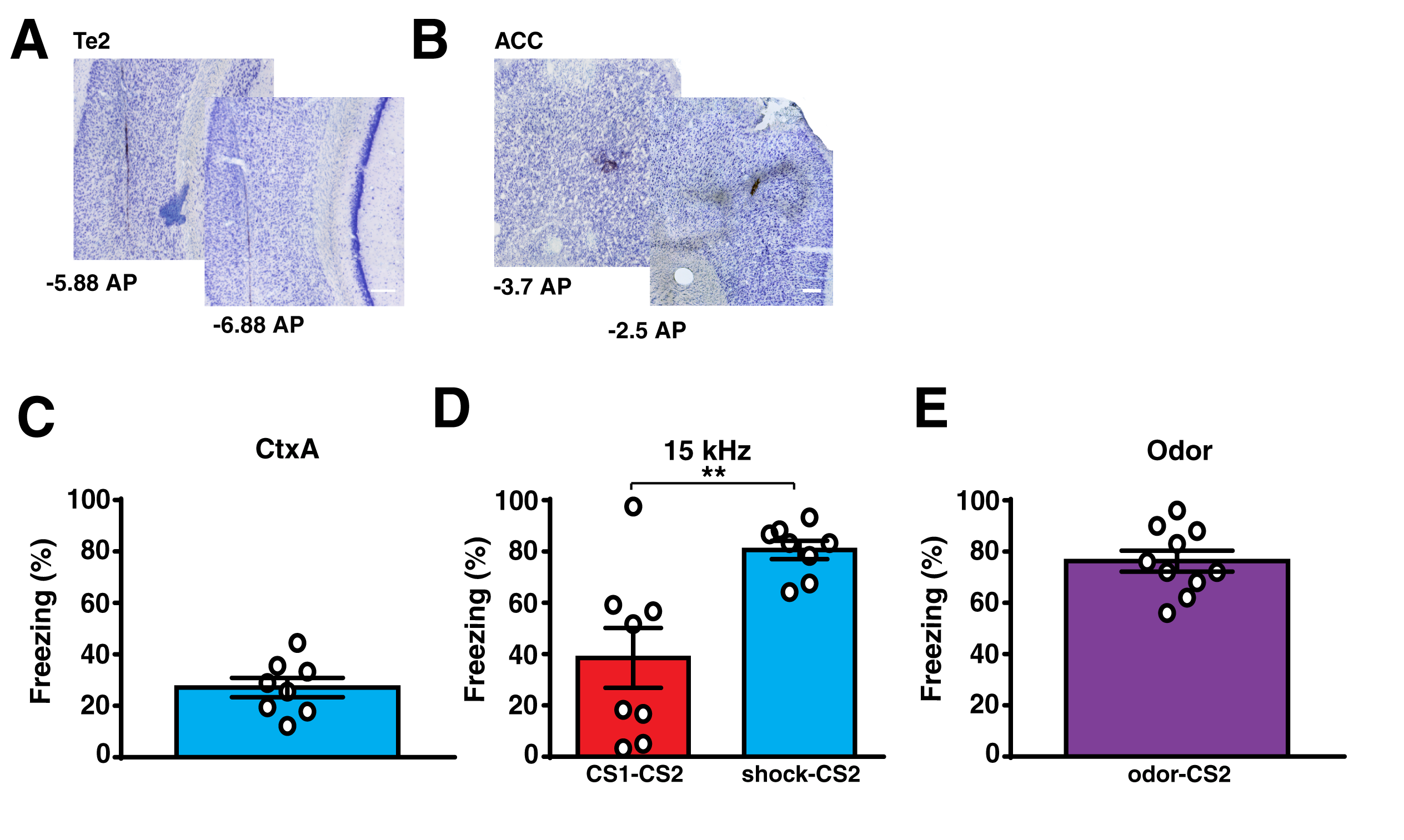

Supplement: S1 Fig — Magnification of the needle tracks of Te2 (A) and ACC cortex (B) injected with CNQX, selected as examples. (C) When represented with the same context 2 weeks after the procedure, shock-only animals showed a low fear response, demonstrating that the procedure did not elicit a conditioned freezing to the context where the shock was delivered. (D) Similar results as in Fig 1 were obtained by counterbalancing the 2 tones employed as CSs (CS1, 3 kHz and CS2, 15 kHz) (Student t test, t(14) = 3.44, p = 0.0040, Glass’s d = 4.14). (E) In the odor-CS conditioned rats freezing to the odor, 2 weeks after conditioning, was high even if tested in a different environment with respect to the conditioning context, thereby showing that fear was specifically associated with this cue delivery. Scale bars, 300 μm. **P < 0.01. All data are mean and SEM. The summary data for S1 Fig can be found in supporting information in the file named S1 Supporting Figure Data. (TIF) [file pbio.3001789.s001.tif]

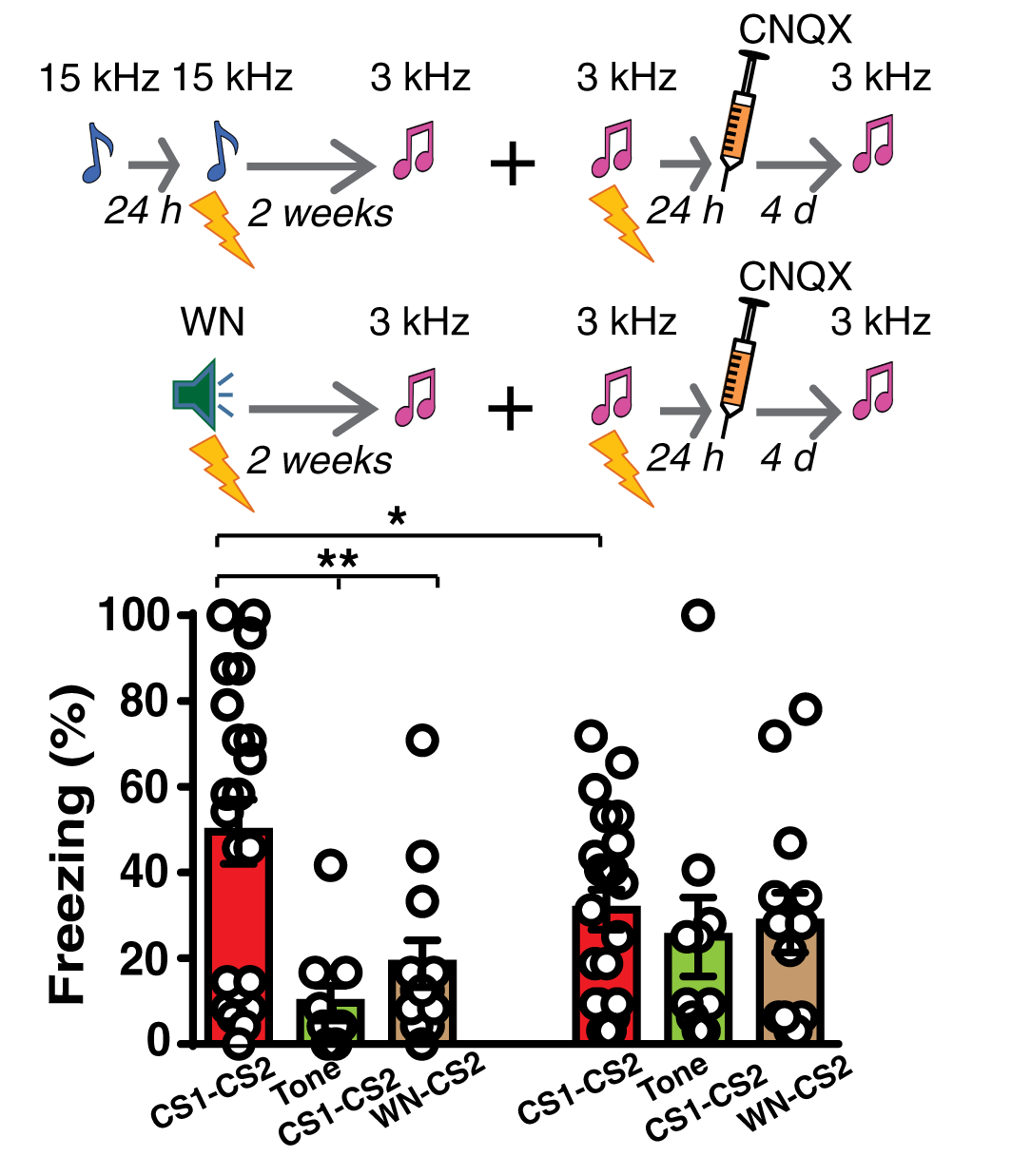

Supplement: S2 Fig — A 3 × 2 mixed-design ANOVA (main effect of group: F(2,42) = 6.478, p = 0.004, η2 = 0. 236, main effect of condition: F(1,42) = 0.161, p = 0.691, η2 = 0.004, group × condition interaction F(2,42) = 3.942, p = 0.027, η2 = 0.158) showed that freezing to the 3 kHz tone before its association to the US was lower in animals that received the 15 kHz tone pre-exposure before the 15 kHz-US pairing (n = 10, p = 0.001) or in rats conditioned to a white noise (n = 13, p = 0.008) as compared to CS1-CS2 animals (n = 22) that showed a variable fear generalization response. However, after CS-US learning and cnqx injections in Te2 cortex, recent fear memory was impaired in all groups (p > 0.05). Simple main effect within groups (before and after CS-US learning followed by cnqx injection): CS1-CS2, p = 0.025; Tone-CS1-CS2, p = 0.189; WN-CS2, p = 0.347) *P < 0.05, **P < 0.01. All data are mean and SEM. The summary data for S2 Fig can be found in Supporting information in the file named S1 Supporting Figure Data. (TIF) [file pbio.3001789.s002.tif]

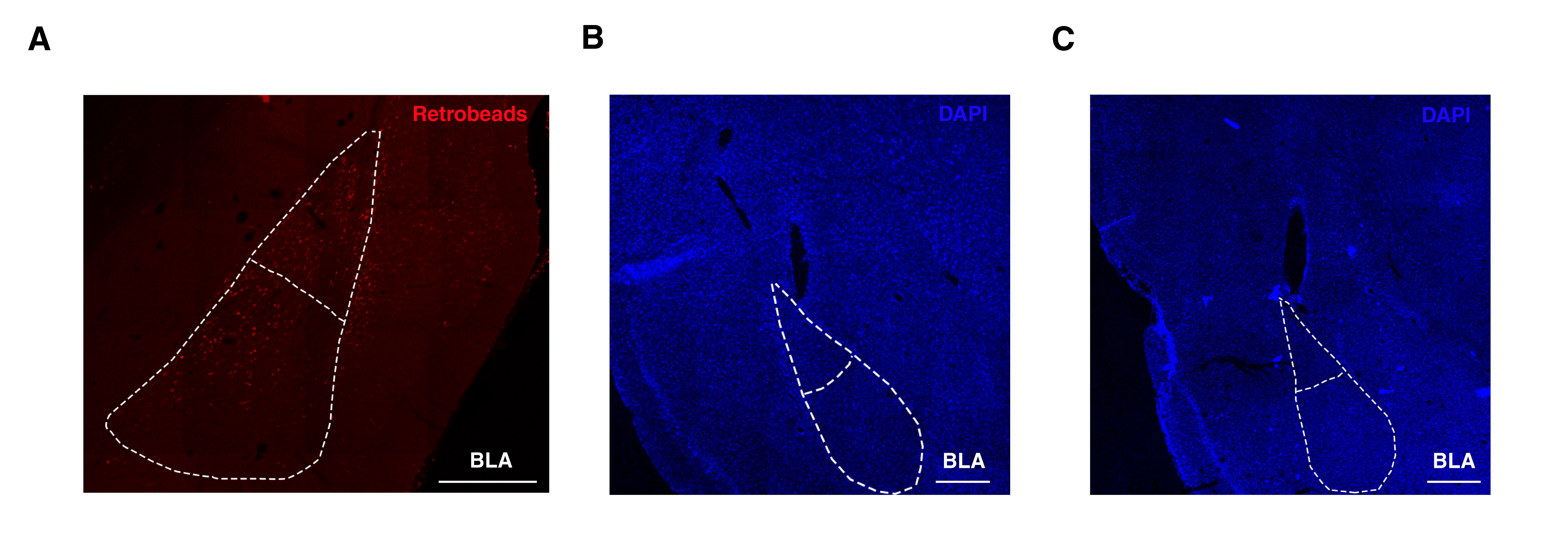

Supplement: S3 Fig — (A) Random example of retrobeads injection targeting BLA. (B and C) Examples of optical fiber placements above the BLA of animals injected with AAV vectors in Te2 (B) and ACC cortex (C). Scale bars, 500 μm. (TIF) [file pbio.3001789.s003.tif]
