# Supplementary material for: Prior fear learning enables the rapid assimilation of new fear memories directly into cortical networks
Source: PLoS Biol. 2022 Sep 30;20(9):e3001789. doi: 10.1371/journal.pbio.3001789 (PMC9555644; doi:10.1371/journal.pbio.3001789)
Supplement: S1 Statistical Test Data — (DOCX) [file pbio.3001789.s010.docx]

| **Figure 1** | **Applied test** | **F and degrees of freedom** | **P value** | **Pairwise comparison** | **N of each group** |
| --- | --- | --- | --- | --- | --- |
| **1C** | one-way ANOVA | F_(3,40)_=4.367 | p = 0.0094 | Tukey: CS1-CS _cnqx_ vs CS1-CS _vehicle_, p = 0.0208;  CS1-CS _cnqx_ vs shock-CS _cnqx_, p = 0.0183;  CS1-CS _cnqx_ vs shock-CS _vehicle_, p = 0.0401; other comparisons, p>0.05 | CS1-CS, _cnqx_ n=10  shock-CS, _cnqx_ n = 8  CS1-CS, _vehicle_ n=15  shock-CS, _vehicle_ n = 11 |
| **1E** | Student’s t test | t_(19)_ = 1.319 | p = 0.2027 | NA | odor-CS, n= 10  Tone-CS, n= 11 |
| **1G** | one-way ANOVA | F_(2,28)_ = 0.80 | p = 0.4554 | NA | CS1-CS 7h, n=11  CS1-CS 24h, n=9  CS1-CS cnqx cnqx, n=11 |
| **1I** | one-way ANOVA | F_(3,49)_=6.99 | p = 0.0005 | Tukey: CS1-CS _cnqx_ vs CS1-CS _vehicle_, p = 0.0046; CS1-CS _cnqx_ vs shock-CS _cnqx_, p = 0.0017; CS1-CS _cnqx_ vs shock-CS _vehicle_, p = 0.0065; other comparisons, p>0.05 | CS1-CS _cnqx_, n = 15  shock-CS _cnqx_, n = 18  CS1-CS _vehicle_, n=12,  shock-CS, _vehicle_ n = 8 |
| **1K** | one-way ANOVA | F_(3,39)_=4.29 | p = 0.0104 | Tukey: CS1-CS _aniso_ vs CS1-CS _vehicle_, p = 0.0443; CS1-CS _aniso_ vs shock-CS _aniso_, p = 0.0180; CS1-CS _aniso_ vs shock-CS _vehicle_, p = 0.0465; other comparisons, p>0.05 | CS1-CS _aniso_, n=12  shock-CS _aniso_ n=11  CS1-CS _vehicle_, n=12  shock-CS, _vehicle_ n = 8 |
| **Figure 2** | **Applied test** | **F and degrees of freedom** | **P value** | **Pairwise comparison** | **N of each group** |
| **2B** | Student’s t-test | t_(16)_ = 0.11 | p = 0.9132 | NA | CS1-CS, n = 9  shock-CS, n = 9 |
| **2C** | Student’s t-test | t_(16)_ = 2.49 | p = 0.0238 | NA | CS1-CS, n = 9  shock-CS, n = 9 |
| **2D** | Student’s t-test | t_(16)_ = 3.05 | p = 0.0076 | NA | CS1-CS, n = 9  shock-CS, n = 9 |
| **2E** | Pearson’s correlation | r = 0.69 | p = 0.0013 | NA | CS1-CS, n = 9  shock-CS, n = 9 |
| **2F** | Student’s t-test | t_(16)_ = 1.59 | p = 0.1302 | NA | CS1-CS, n = 9  shock-CS, n = 9 |
| **Figure 3** | **Applied test** | **F and degrees of freedom** | **P value** | **Pairwise comparison** | **N of each group** |
| **3D** | one-way ANOVA | F_(2,23)_=9.85 | p = 0.0008 | CS1-CS _eNpHR3.0-mCherry_ vs CS1-CS _control-mCherry_, p = 0.0040; CS1-CS _eNpHR3.0-mCherry_ vs shock-CS _eNpHR3.0-mCherry_ , p = 0.0016; CS1-CS _control-mCherry_ vs shock-CS _eNpHR3.0-mCherry_ , p = 0.962 | CS1-CS _eNpHR3.0-mCherry_ n=9  CS1-CS _control-mCherry_ n=8  shock-CS _eNpHR3.0-mCherry_ n=9 |
| **3E** | one-way ANOVA | F_(2,23)_=11.29 | p = 0.0004 | Tukey: CS1-CS _eNpHR3.0-mCherry_ vs CS1-CS _control-mCherry_ , p = 0.0008; CS1-CS _eNpHR3.0-mCherry_ vs shock-CS _eNpHR3.0-mCherry_ , p = 0.972; CS1-CS _control-mCherry_ vs shock-CS _eNpHR3.0-mCherry_, p = 0.0014 | CS1-CS _eNpHR3.0-mCherry_ n=9  CS1-CS _control-mCherry_ n=8  shock-CS _eNpHR3.0-mCherry_ n=9 |
| **Figure 4** | **Applied test** | **F and degrees of freedom** | **P value** | **Pairwise comparison** | **N of each group** |
| **4 C** | one-way ANOVA | F_(3,41)_=5.612 | p = 0.0026 | Tukey: CtxA-CtxB _cnqx_ vs CtxA-CtxB _vehicle_, p = 0.0041; CtxA-CtxB _cnqx_ vs shock-CtxB _cnqx_, p = 0.0108; CtxA-CtxB _cnqx_ vs shock-CtxB _vehicle_, p = 0.0343; other comparisons, p>0.05 | CtxA-CtxB _cnqx_, n =12  shock-CtxB _cnqx_, n = 11  CtxA-CtxB _vehicle_, n =11,  shock-CtxB _vehicle_, n=11 |
| **4 E** | one-way ANOVA | F_(3,37)_ = 1.64 | p = 0.195 | NA | CtxA-CtxB 7h, n=10  CtxA-CtxB 7h, n=11  CtxA-CtxB cnqx cnqx, n=8  Context-CtxB, n= 12 |
| **4 G** | one-way ANOVA | F_(3,38)_=7.32 | p = 0.0005 | Tukey: CtxA-CtxB _cnqx_ vs CtxA-CtxB _vehicle_, p = 0.0227; CtxA-CtxB _cnqx_ vs shock-CtxB _cnqx_, p = 0.0276; CtxA-CtxB _cnqx_ vs shock-CtxB _vehicle_, p = 0.0003; other comparisons, p>0.05 | CtxA-CtxB _cnqx_, n =10  shock-CtxB _cnqx_, n = 13  CtxA-CtxB _vehicle_, n =12  shock-CtxB _vehicle_, n=7 |
| **4 I** | one-way ANOVA | F_(3,35)_=7.820 | p = 0.0004 | Tukey: CtxA-CtxB _aniso_ vs CtxA-CtxB _vehicle_, p = 0.0146; CtxA-CtxB _aniso_ vs shock-CtxB _aniso_, p = 0.0021; CtxA-CtxB _aniso_ vs shock-CtxB _vehicle_, p = 0.0004; other comparisons, p>0.05 | CtxA-CtxB _cnqx_, n =8  shock-CtxB _cnqx_, n = 12  CtxA-CtxB _vehicle_, n =12  shock-CtxB _vehicle_, n=7 |
| **Figure 5** | **Applied test** | **F and degrees of freedom** | **P value** | **Pairwise comparison** | **N of each group** |
| **5D** | one-way ANOVA | F(2,23)=9.90 | p = 0.0008 | Tukey: CtxA-CtxB eNpHR3.0-mCherry vs CtxA-CtxB control-mCherry, p = 0.0055; CtxA-CtxB eNpHR3.0-mCherry vs shock-CtxB eNpHR3.0-mCherry, p = 0.0010; CtxA-CtxB control-mCherry vs shock-CtxB eNpHR3.0-mCherry , p = 0.8629 | CtxA-CtxB _eNpHR3.0-mCherry_ n=8; CtxA-CtxB _control-mCherry_ n=8  shock-CtxB _eNpHR3.0-mCherry_ n=10 |
| **5E** | one-way ANOVA | F_(2,23)_=5.269 | p = 0.0131 | CtxA-CtxB _eNpHR3.0-mCherry_ vs CtxA-CtxB _control-mCherry_ p = 0.0098; CtxA-CtxB _eNpHR3.0-mCherry_ vs shock-CtxB _eNpHR3.0-mCherry_, p = 0.3203; CtxA-CtxB _control-mCherry_ vs shock-CtxB_eNpHR3.0-mCherry_ , p = 0.1521 | CtxA-CtxB _eNpHR3.0-mCherry_ n=8; CtxA-CtxB _control-mCherry_ n=8  shock-CtxB _eNpHR3.0-mCherry_ n=10 |
| **Figure 6** | **Applied test** | **F and degrees of freedom** | **P value** | **Pairwise comparison** | **N of each group** |
| **6C** | one-way ANOVA | F_(3,27)_=23.59 | p < 0.0001 | Tukey: CtxA-CtxB _nmda_ vs CtxA-CtxB _sham,_ p < 0.0001; CtxA-CtxB _nmda_ vs shock-CtxB _nmda_, p = 0.2805; CtxA-CtxB _nmda_ vs shock-CtxB _sham_, p = 0.0001; CtxA-CtxB _sham_ vs shock-CtxB _nmda_, p < 0.0001 ; CtxA-CtxB _sham_ vs shock-CtxB _sham_, p = 0.9776; shock-CtxB _nmda_ vs shock-CtxB _sham_, p < 0.0001 | CtxA-CtxB _nmda_, n=11  shock-CtxB _nmda_, n=7  CtxA-CtxB _sham_, n=8  shock-CtxB _sham_, n=5 |
| **6E** | one-way ANOVA | F_(3,23)_=11.24 | p < 0.0001 | Tukey: CtxA-CtxB _cnqx_ vs CtxA-CtxB _vehicle_ p = 0.0078; CtxA-CtxB _cnqx_ vs shock-CtxB _cnqx_, p = 0.7442; CtxA-CtxB_cnqx_ vs shock-CtxB_vehicle_, p = 0.0054; CtxA-CtxB _vehicle_ vs shock-CtxB _cnqx_, p = 0.0012 ; CtxA-CtxB _vehicle_ vs shock-CtxB _vehicle_, p = 0.9992; shock-CtxB _cnqx_ vs shock-CtxB _vehicle_, p = 0.0008 | CtxA-CtxB _cnqx_, n=9  shock-CtxB _cnqx_, n=8  CtxA-CtxB _vehicle_, n=5  shock-CtxB _vehicle_, n=5 |
| **6G** | one-way ANOVA | F_(3,31)_=9.06 | p = 0.0002 | Tukey: CtxA-CtxB _nmda_ vs CtxA-CtxB _sham_ p = 0.8383; CtxA-CtxB _nmda_ vs shock-CtxB _nmda_, p = 0.012; CtxA-CtxB_nmda_ vs shock-CtxB _sham_, p = 0.5841; CtxA-CtxB _sham_ vs shock-CtxB _nmda_, p = 0.0056 ; CtxA-CtxB _sham_ vs shock-CtxB _sham_, p = 0.9927 ; shock-CtxB _nmda_ vs shock-CtxB _sham_, p = 0.0004 | CtxA-CtxB _nmda_, n=9  shock-CtxB _nmda_, n=13  CtxA-CtxB _sham_, n=5  shock-CtxB _sham_, n=8 |
| **S1B** | Student’s t-test | t_(14)_ = 3.44 | p = 0.0040 | NA | CS1-CS _cnqx_, n=8  shock-CS _cnqx_, n = 8 |
| **S2** | A 3 × 2 mixed-design ANOVA | main effect of group: F_(2,42)_  main effect of condition: F_(1,42)_ = 0.161  , group × condition interaction F_(2,42)_ = 3.942, | p = 0.004  p = 0.691  p = 0.027 | Between variable  Tone-CS1-CS vs CS1-CS= 0.001;  WN-CS vs CS1-CS= p=0.008  Within variable  CS1-CS, p *=* 0.025; Tone-CS1-CS, p*=* 0.189; WN-CS, p = 0.347 | Tone-CS1-CS, n=10;  CS1-CS, n=22  WN-CS, n=13 |
