# Supplementary material for: Prior fear learning enables the rapid assimilation of new fear memories directly into cortical networks
Source: PLoS Biol. 2022 Sep 30;20(9):e3001789. doi: 10.1371/journal.pbio.3001789 (PMC9555644; doi:10.1371/journal.pbio.3001789)
Supplement: S1 Table — (DOCX) [file pbio.3001789.s012.docx]

**S1 Table**

| **Figures** | **Group** | **Exp 1** | **Exp 2** | **Exp 3** | **Exp 4** | **Exp 5** | **Exp 6** | **Included** | **Excluded** |
| --- | --- | --- | --- | --- | --- | --- | --- | --- | --- |
| **Fig 1C** | CS1-CS2 cnqx | n=4 | n=3 | n=3 | n=2 |  |  | n=10 | n=2 |
|  | shock-CS2 cnqx | n=3 | n=3 | n=2 | n=1 |  |  | n=8 | n=1 |
|  | CS1-CS2 vehicle |  |  |  | n=4 | n=6 | n=6 | n=15 | n=1 |
|  | shock-CS2 vehicle |  |  |  | n=3 | n=6 | n=4 | n=11 | n=2 |
| **Fig 1E** | Odor-CS2 | n=4 | n=4 | n=2 |  |  |  | n=10 | n=0 |
|  | Tone-CS2 | n=4 | n=4 | n=4 |  |  |  | n=11 | n=1 |
| **Fig 1G** | CS1-CS2 7h | n=4 | n=4 | n=3 |  |  |  | n=11 | n=0 |
|  | CS1-CS2 24h | n=3 | n=3 | n=3 |  |  |  | n=9 | n=0 |
|  | CS1-CS2 cnqx-cnqx | n=4 | n=4 | n=4 |  |  |  | n=11 | n=1 |
| **Fig 1I** | CS1-CS2 cnqx | n=7 | n=7 | n=2 |  |  |  | n=15 | n=1 |
|  | shock-CS2 cnqx | n=8 | n=8 | n=4 |  |  |  | n=18 | n=2 |
|  | CS1-CS2 vehicle |  |  | n=4 | n=4 | n=4 |  | n=12 | n=0 |
|  | shock-CS2 vehicle |  |  | n=2 | n=4 | n=3 |  | n=8 | n=1 |
| **Fig 1K*** | CS1-CS2 aniso | n=8 | n=4 |  |  |  |  | n=12 | n=0 |
| *As detailed in the Fig .1J-K legend, these groups were compared with the same control groups as in Fig. 1I | shock-CS2 aniso | n=8 | n=4 |  |  |  |  | n=11 | n=1 |
| **Fig 2B-2F** | CS1-CS2 | n=4 | n=4 | n=3 |  |  |  | n=9 | n=2 |
|  | shock-CS2 | n=4 | n=3 | n=3 |  |  |  | n=9 | n=1 |
| **Fig 3D,E** | CS1-CS2 eNpHR3.0 | n=5 | n=4 | n=2 |  |  |  | n=9 | n=2 |
|  | shock-CS2  eNpHR3.0 | n=4 | n=4 | n=2 |  |  |  | n=9 | n=1 |
|  | CS1-CS2-AAV control |  |  | n=2 | n=7 |  |  | n=8 | n=1 |
| **Fig 4C** | CtxA-CtxB cnqx | n=6 | n=5 | n=4 |  |  |  | n=12 | n=3 |
|  | shock-CtxB cnqx | n=5 | n=4 | n=3 |  |  |  | n=11 | n=1 |
|  | CtxA-CtxB vehicle |  |  | n=3 | n=6 | n=4 |  | n=11 | n=2 |
|  | shock-CtxB vehicle |  |  | n=3 | n=4 | n=6 |  | n=11 | n=2 |
| **Fig 4E** | CtxA-CtxB 7h | n=4 | n=4 | n=3 |  |  |  | n=10 | n=1 |
|  | CtxA-CtxB 24h | n=5 | n=5 | n=4 |  |  |  | n=11 | n=3 |
|  | CtxA-CtxB cnqx-cnqx | n=4 | n=4 | n=2 |  |  |  | n=8 | n=2 |
|  | Context-CtxB | n=5 | n=5 | n=4 |  |  |  | n=12 | n=2 |
| **Fig 4G** | CtxA-CtxB cnqx | n=6 | n=5 | n=2 |  |  |  | n=10 | n=3 |
|  | shock-CtxB cnqx | n=7 | n=5 | n=3 |  |  |  | n=13 | n=2 |
|  | CtxA-CtxB vehicle |  |  | n=3 | n=6 | n=5 |  | n=12 | n=2 |
|  | shock-CtxB vehicle |  |  | n=3 | n=4 | n=3 |  | n=7 | n=3 |
| **Fig 4I** | CtxA-CtxB aniso | n=6 | n=5 |  |  |  |  | n=8 | n=3 |
| *As detailed in the Fig .4H-I legend, data from Anisomycin-injected rats were compared with the same control groups as in Fig. 4G | shock-CtxB aniso | n=7 | n=7 |  |  |  |  | n=12 | n=2 |
| **Fig 5D,E** | CtxA-CtxB eNpHR3.0 | n=5 | n=4 | n=2 |  |  |  | n=8 | n=3 |
|  | shock-CtxB  eNpHR3.0 | n=5 | n=4 | n=2 |  |  |  | n=10 | n=1 |
|  | CtxA-CtxB AAV control |  |  | n=2 | n=8 |  |  | n=8 | n=2 |
| **Fig 6C** | CtxA-CtxB nmda | n=5 | n=5 | n=4 |  |  |  | n=11 | n=3 |
|  | shock-CtxB nmda | n=4 | n=2 | n=2 |  |  |  | n=7 | n=1 |
|  | CtxA-CtxB sham |  |  | n=3 | n=5 |  |  | n=8 | n=0 |
|  | shock-CtxB sham |  |  | n=1 | n=4 |  |  | n=5 | n=0 |
| **Fig 6E** | CtxA-CtxB cnqx | n=4 | n=2 | n=3 |  |  |  | n=9 | n=0 |
|  | shock-CtxB cnqx | n=3 | n=3 | n=3 |  |  |  | n=8 | n=1 |
|  | CtxA-CtxB vehicle |  |  | n=2 | n=5 |  |  | n=5 | n=2 |
|  | shock-CtxB vehicle |  |  | n=2 | n=4 |  |  | n=5 | n=1 |
| **Fig 6G** | CtxA-CtxB nmda | n=4 | n=4 | n=2 |  |  |  | n=9 | n=1 |
|  | shock-CtxB nmda | n=5 | n=5 | n=5 |  |  |  | n=13 | n=2 |
|  | CtxA-CtxB sham |  |  | n=2 | n=3 |  |  | n=5 | n=0 |
|  | shock-CtxB sham |  |  | n=3 | n=5 |  |  | n=8 | n=0 |
| **Fig S1D** | CS1-CS2 | n=4 | n=2 | n=2 |  |  |  | n=8 | n=0 |
|  | shock-CS2 | n=4 | n=2 | n=2 |  |  |  | n=8 | n=0 |
| **Fig S2** | CS1-CS2 | n=10 | n=9 | n=9 |  |  |  | n=22 | n=6 |
|  | Tone-CS1-CS2 | n=5 | n=4 | n=3 |  |  |  | n=10 | n=2 |
|  | WN-CS2 | n=5 | n=5 | n=4 |  |  |  | n=13 | n=1 |
